# Macromolecular crowding regulates matrix composition and gene expression in human gingival fibroblast cultures

**DOI:** 10.1038/s41598-023-29252-1

**Published:** 2023-02-04

**Authors:** Rajesvaran Ramalingam, Guoqiao Jiang, Hannu Larjava, Lari Häkkinen

**Affiliations:** Department of Oral Biological and Medical Sciences, Faculty of Dentistry, University of British Columbia, 2199 Wesbrook Mall, Vancouver, BC V6T 1Z3 Canada

**Keywords:** Cell adhesion, Cell growth, Cell signalling, Skin models, Biological techniques, Cell biology, Biotechnology, Assay systems, Regenerative medicine, Tissue engineering

## Abstract

Standard cell cultures are performed in aqueous media with a low macromolecule concentration compared to tissue microenvironment. In macromolecular crowding (MMC) experiments, synthetic polymeric crowders are added into cell culture media to better mimic macromolecule concentrations found in vivo. However, their effect on cultured cells is incompletely understood and appears context-dependent. Here we show using human gingival fibroblasts, a cell type associated with fast and scarless wound healing, that MMC (standard medium supplemented with Ficoll 70/400) potently modulates fibroblast phenotype and extracellular matrix (ECM) composition compared to standard culture media (nMMC) over time. MMC significantly reduced cell numbers, but increased accumulation of collagen I, cellular fibronectin, and tenascin C, while suppressing level of SPARC (Secreted Protein Acidic and Cysteine Rich). Out of the 75 wound healing and ECM related genes studied, MMC significantly modulated expression of 25 genes compared to nMMC condition. MMC also suppressed myofibroblast markers and promoted deposition of basement membrane molecules collagen IV, laminin 1, and expression of *LAMB3 (Laminin Subunit Beta 3)* gene. In cell-derived matrices produced by a novel decellularization protocol, the altered molecular composition of MMC matrices was replicated. Thus, MMC may improve cell culture models for research and provide novel approaches for regenerative therapy.

## Introduction

Extracellular matrix (ECM) niche of cells in tissues is three-dimensional (3D) and composed of various structural ECM components, such as collagen, proteoglycans, glycoproteins and glycosaminoglycans, and signaling molecules, including growth factors and cytokines^1^. Composition of the ECM niche is specific for distinct fibroblast subpopulations found in different tissues^2^. Interactions of cells with the ECM niche provides biochemical and mechanical cues that are critical for determination of cell identity, state and function during development, homeostasis, repair and pathological conditions^1^. The ECM niche in tissues is typically crowded with various molecules, which modulates their diffusion, conformation, and interactions with each other and cells^3,4^. While traditional cell cultures in aqueous media have provided tremendous amount of important knowledge about various aspects of cell phenotype and functions and allowed us to develop tools for regenerative therapy, cells in these models are often kept in two-dimensional monolayer cultures in a non-crowded environment. For instance, mesenchymal stem cells in bone marrow are embedded in a three-dimensional ECM niche that has an estimated concentration of 21–80 mg/mL of macromolecules, while in standard cell culture medium their concentration is only about 4–16 mg/mL if serum concentration is 5–20%^3,5,6^. Therefore, cell culture models that better mimic the dimensionality and macromolecular crowding of the natural ECM niche found in vivo have been introduced. In particular, the inclusion of synthetic macromolecular crowders (MMCs), such as Ficoll, dextran, polyethylene glycol, and polyvinyl alcohol, into culture media have been proposed to compensate for this deficiency^7^. Recent evidence from mass spectrometry-based proteomics of adult human dermal fibroblast ECMs from cultures generated using MMC (Ficoll 70/400) and information from public proteomics databases of core matrisome proteins (collagens, glycoproteins, and proteoglycans) from adult human skin support the idea that MMC generates matrices with in vivo-like composition^8^. Furthermore, MMC has allowed generation of cell cultures that are composed of layers of cells embedded in their own ECM niche (three-dimensionality) in an accelerated rate^9^. Therefore, MMC is emerging as a technology to generate cell culture conditions that may better mimic the ECM niche of cells in tissues than the traditional methods. Use of MMCs may not only benefit research about interactions of cells with their ECM niche, but could also provide better tissue engineering-based modalities for translational research and regenerative therapy. Interestingly, however, the effect of MMC on cultured cells appears to vary based on cell type and tissue origin, culture conditions, and MMCs used^7^. Thus, effect of MMC appears cell-type specific and may need to be optimized for different cellular contexts.

Fibroblasts are the key cells that generate the ECM niche in various soft connective tissues, including skin and oral mucosal gingiva. Unlike mostly mesoderm-derived skin fibroblasts, gingival fibroblasts originate from the neural crest. In addition, they associate functionally with fast regenerative-like wound healing, akin to fetal skin wound healing, compared to adult scar-forming skin. In addition, gingival fibroblasts display distinct gene expression repertoire in vivo and in vitro compared to skin fibroblasts^10–15^. For instance, at protein level, cultured gingival fibroblasts show differences in abundance, organization, structure, and composition of proteins that are important in creating a tissue-specific ECM niche compared to skin fibroblasts^16,17^. These include ECM proteins, integrins that bind the ECM proteins, growth factors that bind and modulate ECM deposition (*e.g.,* TGF-β1 and VEGFA), and MMPs that remodel the ECM. In addition, cultured gingival and skin fibroblasts show significant differences in the expression of a number of genes for various ECM niche-associated genes, including ECM molecules, cell surface receptors, MMPs, and growth factors^16–18^. Therefore, gingival fibroblasts may provide a distinct cell type that will allow us to interrogate cell-ECM niche interactions that are important in tissue regeneration and provide opportunities to develop novel tissue engineering modalities for regenerative therapy. However, whether MMC may regulate their phenotype and function is not known.

Among the molecules used for MMC, Ficoll is a neutral, highly branched, hydrophilic polysaccharide formed by the reaction of sucrose and epichlorohydrin, and dissolves well in aqueous solutions ^19,20^. Unlike other charged crowding agents, Ficoll does not easily bind to biological molecules allowing it to occupy space without “scavenging” these molecules from the solution. It is commonly synthesized in molecular weights of 70 kDa (Ficoll 70) and 400 kDa (Ficoll 400) with hydrodynamic radii of 40 Å and 80 Å, respectively ^5^. Therefore, in the present study, we wanted to find out whether culture of human gingival fibroblasts in medium supplemented with MMCs (Ficoll 70/400) modulates their phenotype, composition of ECM niche, and gene expression as compared to cells cultured in similar medium without MMC (nMMC) over time.

## Materials and methods

### Isolation of primary human gingival fibroblasts

The gingival fibroblast strain (GFBL-NLd) used in the study comes from a collection in our laboratory. The tissue biopsy from where the cells were cultured from was collected from a tissue discard obtained during a small routine dental operation of a clinically healthy tissue from a healthy 14-year-old female as previously described^21^. The study was approved by the Clinical Research Ethics Board at the University of British Columbia for all procedures involving human tissues and cells (protocol C05-0047-C02-0076). Informed written consent was obtained from the donor and parents according to the 1975 Declaration of Helsinki. GFBLs were routinely cultured in the standard growth media consisting of Dulbecco’s Modified Eagle’s Medium (DMEM) (CAT#31600-083; Gibco Life Technologies, Grand Island, NY, USA), 10% fetal bovine serum (FBS) (CAT#12484-028; Gibco Life Technologies), and 1% antibiotic/antimycotic (AXB) (CAT#15240-062; Gibco Life Technologies) at 37 °C and 5% CO_2_. Experiments were performed at passages 4–10.

### Three-dimensional (3D) culture of gingival fibroblasts

GFBLs were cultured in the above culture medium until 90% confluency. Cells were then trypsinized and seeded in the standard medium at a density of 25 × 10^3^ cells per cm^2^ on 6-well tissue culture plates (CAT#353046; Corning, Corning, NY, USA) or gelatin coated glass coverslips (CAT#12-545-80; Fisher Scientific, Pittsburgh, PA, USA) placed in 24-well tissue culture plates (CAT#3524; Corning Costar, Kennebunk, ME, USA). After 24 h, medium was replaced with the above medium supplemented with 50 µg/ml ascorbic acid (Millipore Sigma: A-4034, Burlington, MA, USA) (nMMC, control medium) or with 50 µg/ml ascorbic acid and 37.5 mg/mL of Ficoll 70 (F2878; Millipore Sigma) and 25 mg/mL of Ficoll 400 (F4375; Millipore Sigma) (MMC, test medium)^5^. Cells were cultured up to 14 days to generate 3D cultures where cells are embedded in their own ECM^22,23^. Cultures incubated in their normal growth medium for 24 h were designated as day 0 samples. Samples switched to the nMMC or MMC media after the above 24-h period were further cultured for the indicated time before sample collection (designated as day 3, 7, 10, and 14 cultures).

### Real-time RT-PCR (RT-qPCR)

At indicated time points of culture, total RNA was extracted from the cultures with Invitrogen PureLink RNA Mini Kit (CAT#12183018A; ThermoFisher Scientific, Waltham, MA, USA) according to the manufacturer’s instructions and assessed for quantity and purity using the GeneQuant Pro RNA/DNA Calculator (CAT#10619; Amersham Biosciences, Little Chalfont, Buckinghamshire, UK). Total RNA was reverse transcribed with Applied Biosystems High-Capacity cDNA Reverse Transcription Kit (CAT#4308228; ThermoFisher Scientific) according to the manufacturer’s instructions. cDNA was synthesized with a Mastercycler Gradient Reverse-Transcriptase PCR Instrument (CAT#5331; Eppendorf, Hamburg, Germany) with the following program: 1 cycle at 25 °C for 10 min, 1 cycle at 37 °C for 120 min, and 1 cycle at 85 °C for 5 min to heat inactivate the reverse transcriptase. The primers used for RT-qPCR are listed in Supplementary Table S1. Primer design has been described in detail previously^24^. cDNA from each sample was diluted (1.8 ng/mL) and run such that the Ct values were well within the range of their standard curves. Diluted cDNA (5 μL) was mixed with 10 μL of Applied Biosystems Power SYBR Green PCR Master Mix (CAT#4367659; ThermoFisher Scientific) and 5 pM of primers, for a final volume of 20 μL. RT-qPCR amplification was performed on the CFX96 System (Bio-Rad Laboratories, Hercules, CA, USA). At least 2 reference genes were used in reactions performed as a triplicate for each sample with non-transcribed RNA used as a negative control. Data was analyzed according to the comparative Ct method (CFX Manager 2.1; Bio-Rad Laboratories). Statistical comparisons were performed using Log2 transformed values. Results reflect three to seven biological replicates.

### Generation of cell-free cell-derived matrices (CDMs)

In order to develop a novel, efficient decellularization method for the 3D cultures, we tested the use of latrunculin B as an actin destabilizing agent, deoxycholate as a mild detergent, and DNase in sequential incubations to remove cellular elements, including cytoskeletal actin and β-tubulin and DNA from the cultures^25,26^. To this end, GFBLs were cultured for 7 days in their nMMC culture medium. Cultures were then incubated at various concentrations (0.2–5 μM) and times (1–4 h) of latrunculin B (CAT#L-5288; Sigma Aldrich, St. Louis, MO, USA) and 1 × EDTA free protease inhibitor (CAT#11873580001; Roche Diagnostics, Indianapolis, IN, USA) in DMEM at 37 °C^25^. Cultures were washed twice with PBS and incubated in 0.05 M Tris–HCl (pH = 8), 0.15 M NaCl, 1 mM MgCl_2_, 1.0 mM CaCl_2_, and 0.5% sodium deoxycholate (CAT#0613; VWR, Solon, OH, USA) in ultra-pure water at 4 °C for 20 minutes^26^. Cultures were then washed once with PBS and incubated in 10 mM Tris–HCl (pH = 7), 2.5 mM MgCl_2_, 2.5 mM CaCl_2_, and 20 μg/mL DNase (CAT#3778-0100, Akron Biotech, Sarasota, FL, USA) in ultra-pure water at 37 °C for 20 min to remove DNA^26^. Phase contrast microscopy, staining of DNA by DAPI, and immunostaining of actin and β-tubulin (see below) showed that incubation with 0.6 μM latrunculin B for 3 h followed by treatment with deoxycholate and DNase were effective in removing all cellular and cytoskeletal elements and DNA from the cultures. In all subsequent experiments, GFBLs were cultured in the test media for 3, 7, 10, and 14 days before decellularization using 0.6 μM latrunculin B treatment for 3 h before deoxycholate and DNase treatment as above to render cell-free fibroblast-derived 3D ECMs (cell-derived matrices, CDMs).

### Quantification of total protein content in fibroblast cultures and cell-derived matrices

To assess total protein amounts, 3-, 7-, 10- and 14-day cultures (with cells) and parallel CDMs generated in 6-well tissue culture plates were used. GFBL cultures and CDMs were washed with PBS and solubilized in 2% SDS containing 0.005% bromophenol blue and 10% glycerol in 50 mM Tris–HCl (pH = 6.8). Samples were collected by using a rubber policeman, pipetted into homogenizer columns (CAT#HCR003; Omega Bio-Tek, Norcross, GA, USA), and spun at 10,000×*g* for 30 s. Total protein abundance was quantified using the Bio-Rad DC protein assay reagent kit (CAT# 5000111, Bio-Rad Laboratories) and BioRad spectrophotometer (Bio-Rad Laboratories) according to manufacturer's instructions. Analyses for each sample was performed in triplicate. Results reflect a minimum of six biological replicates each with up to 6 parallel wells.

### Gelatin coating of glass coverslips

In order to promote cell adhesion, coverslips were coated with gelatin^27^. To this end, glass coverslips (CAT#12-545-80; Fisher Scientific) were treated overnight in 16 N nitric acid. Nitric acid was removed and the beaker was replenished with Milli-Q 18.2 Ω cm ultra-pure water (Millipore Sigma, Burlington, MA, USA). Every 3 h, existing ultra-pure water was replaced with new ultra-pure water for a total of 10 times. Individual coverslips were rinsed an additional 10 times with ultra-pure water, dried, and autoclaved. Sterile coverslips were placed in 2 cm^2^ sized tissue culture plate wells (CAT#3524; Corning Costar, Kennebunk, ME, USA), washed with PBS, incubated with 0.2% gelatin in PBS at 37 °C for 60 min, washed three times with PBS, crosslinked with 1% glutaraldehyde (CAT#A10500; Alfa Aesar, Ward Hill, MA, USA) in PBS at room temperature for 30 min, followed by three rinses in PBS. Coated coverslips were stored in PBS at + 4 °C for use within one week.

### Immunostaining of the cultures and cell-derived matrices

For immunostaining, 0-, 3-, 7-, 10- and 14-day GFBL cultures generated on gelatin coated glass coverslips (with cells) and parallel CDMs were washed once with PBS, fixed with 4% formaldehyde at room temperature for 20 min, washed three times with PBS, and stored at 4 °C in PBS until immunostained. For immunostaining, cells were permeabilized with 0.5% Triton X-100 in PBS for 4 min. Samples were then washed three times with PBS followed by blocking with PBS + (containing Ca^2+^ and Mg^2+^) containing 10 mg/mL BSA and 1 mg/mL glycine at room temperature for 30 min. Samples were then incubated with a primary antibody (Supplementary Table S2) diluted in 1 mg/mL BSA in PBS in a humidified chamber at 4 °C overnight. The samples were then washed with PBS containing 1 mg/mL BSA and 0.01% Triton X-100 and then incubated with an appropriate Alexa-conjugated secondary antibody (1:200 dilution; Alexa 488/594; Thermo Fisher) at room temperature for 1 h. Nuclei were then stained with 300 nM DAPI (Thermo Fisher) in PBS for 10 min. Samples were mounted with Immu-Mount™ solution (Thermo Fisher) and examined using a Nikon Eclipse 80i Compound Fluorescent Microscope (Nikon Corporation, Tokyo, Japan) and images were captured using Nikon NIS-Elements Basic Research 4.2 software (Nikon Corporation).

### Measurement of nuclear angle deviation, surface area, and aspect ratio

In order to assess cell alignment/orientation in the cultures, mean of standard deviation of the angles of long axis of DAPI-stained nuclei relative to a standardized reference point in each image was measured using Fiji (ImageJ2) software (version 2.3.0/153q; http://imagej.net). In order to quantify cell morphology, nuclear surface area, as a measure of cell size, and aspect ratio, as a measure of cell elongation^28–31^, were calculated. To this end, nuclei were outlined using the polygon selection tool and surface areas and aspect ratios were calculated using the measure function in the software. For the above measurements, standardized fluorescence microscope images taken with × 20 objective using stratified random sampling within the middle-third area between the center and periphery of each coverslip were used. Measurements represent in total of about 20–100 cells per image in triplicate for each coverslip from three biological replicates at each time point. Results are presented as mean + /− SEM for all the measurements.

### Cell count determination

In order to quantify cell numbers in the cultures generated on coverslips, number of DAPI-stained nuclei were counted manually using the Fiji software. To this end, standardized fluorescence microscope images were taken randomly with × 20 objective within the middle-third area between the center and periphery of each coverslip. In total seven images per sample were obtained representing 100–600 cells per image depending on time point. Mean + /− SEM from four repeated experiments were calculated for data presentation.

### Quantitation of protein abundance in immunostained samples

In order to assess protein abundance in the immunostained samples, minimum of three standardized fluorescence microscope images were obtained using stratified random sampling as above. Integrated fluorescence density/intensity (the sum of fluorescence intensity from each pixel of each image) was calculated using the measure function in the Fiji software as a measure of protein abundance. For comparison of staining intensity for a given molecule over time, relative total fluorescence density/intensity and relative fluorescence density/intensity per cell was calculated by normalizing values to those obtained from Day 0 samples. For pairwise comparison between cultures generated in different test media at day 14, relative immunostaining density/intensity for a given molecule normalized for one of the test treatments was calculated. To assess relative abundance of αSMA stress fibers, standardized thresholding function in the Fiji software was applied to each image to exclude staining not associated with stress fibers before calculating integrated fluorescence density/intensity per cell as above (Supplementary Figure S1). Results are presented as mean + /− SEM from 6 to 12 measurements from two repeated experiments.

### Western blotting

Western blotting was performed as previously described^16^. Briefly, cultures were washed once with PBS, lysed in 1 × Laemmli sample buffer, collected using a rubber policeman, and filtered through homogenizer mini columns and total protein concentration determined as above. Equal amount of protein of each sample with 2-mercaptoethanol (5%) was separated by SDS/PAGE (7.5%) and transferred onto Hybond Protran membrane (Amersham). The membranes were incubated in Odyssey Blocking Buffer (LI-COR Biosciences, Lincoln, NE) for 1 h, followed by incubation with anti-αSMA primary antibody (Supplementary Table S2) overnight at + 4 °C. After incubation with species-appropriate IRdye-conjugated secondary antibodies (LI-COR) for 1 h, the blots were washed and scanned with LI-COR Odyssey Infrared Imaging system. The results were quantified using ImageJ software (version V1.53U; http://imagej.net).

### Statistical analysis

Data is presented as mean ± SEM with statistical analysis performed using IBM SPSS Software Version 23. Number of independent experiments (n) are indicated in figure legends. Normality of the data and equality of variance was tested using Shapiro–Wilk test and Levene’s test, respectively. Statistical comparisons were performed using independent samples t-test (two groups and normally distributed) or one-way ANOVA (greater than two groups and data normally distributed) followed by either Tukey HSD (when variance is equal) or Games-Howell (when variance is unequal) post hoc test.

## Results

### MMC modulates gingival fibroblast numbers and orientation

**Figure 1:**
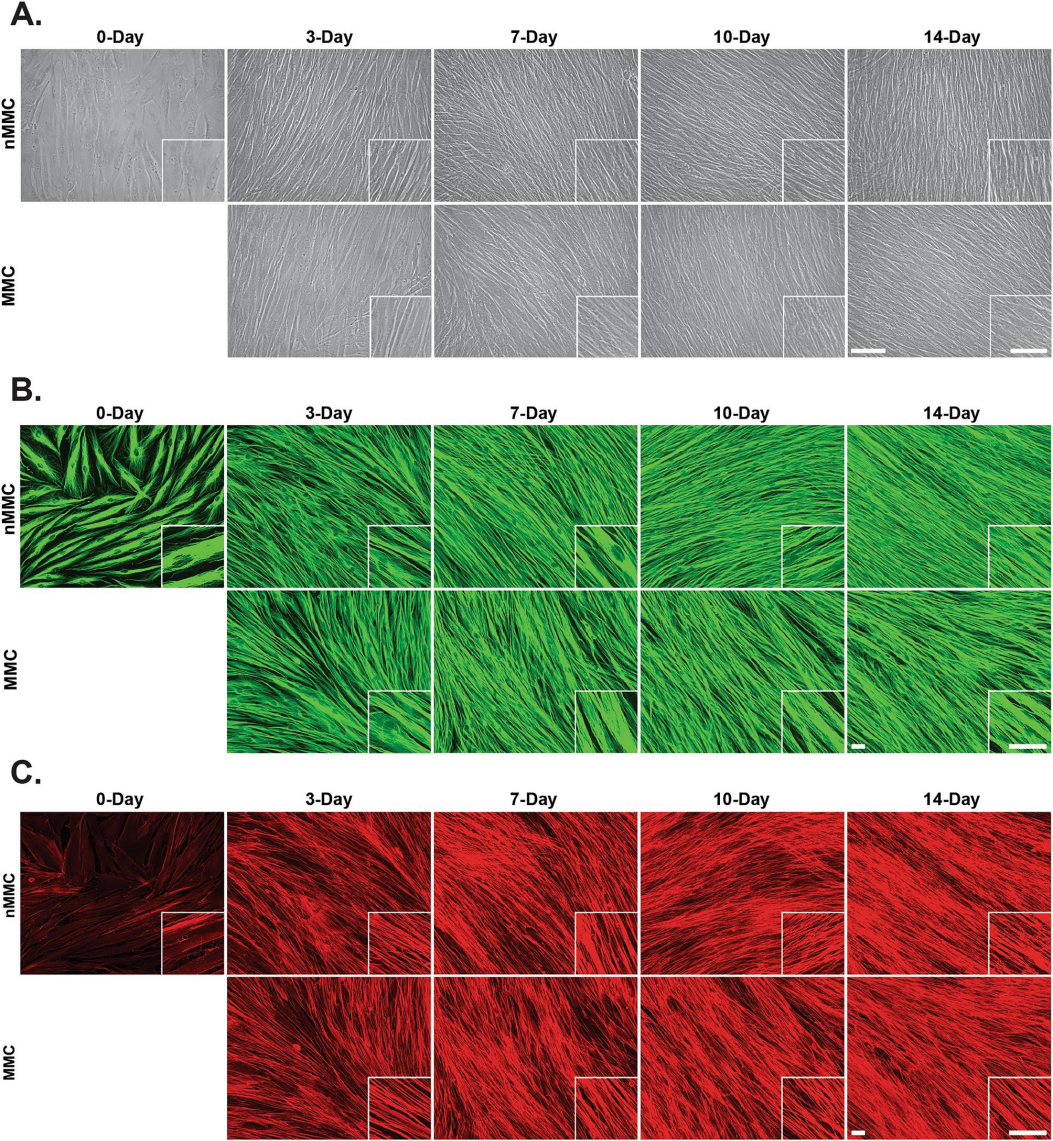
Characterization of fibroblast MMC and nMMC cultures by phase contrast microscopy and immunostaining of cytoskeletal β-tubulin and actin over time. Representative phase contrast microscope (**A**), immunostaining of β-tubulin (**B**), and staining of total fibrillar actin with fluorescently labelled phalloidin (**C**) images from 6 to 12 standardized images obtained from each time point and treatment from two repeated experiments are shown. Inserts show higher magnification of select areas. Images in inserts are individually optimized for brightness/contrast. Magnification bars = 40 μm.

**Figure 2:**
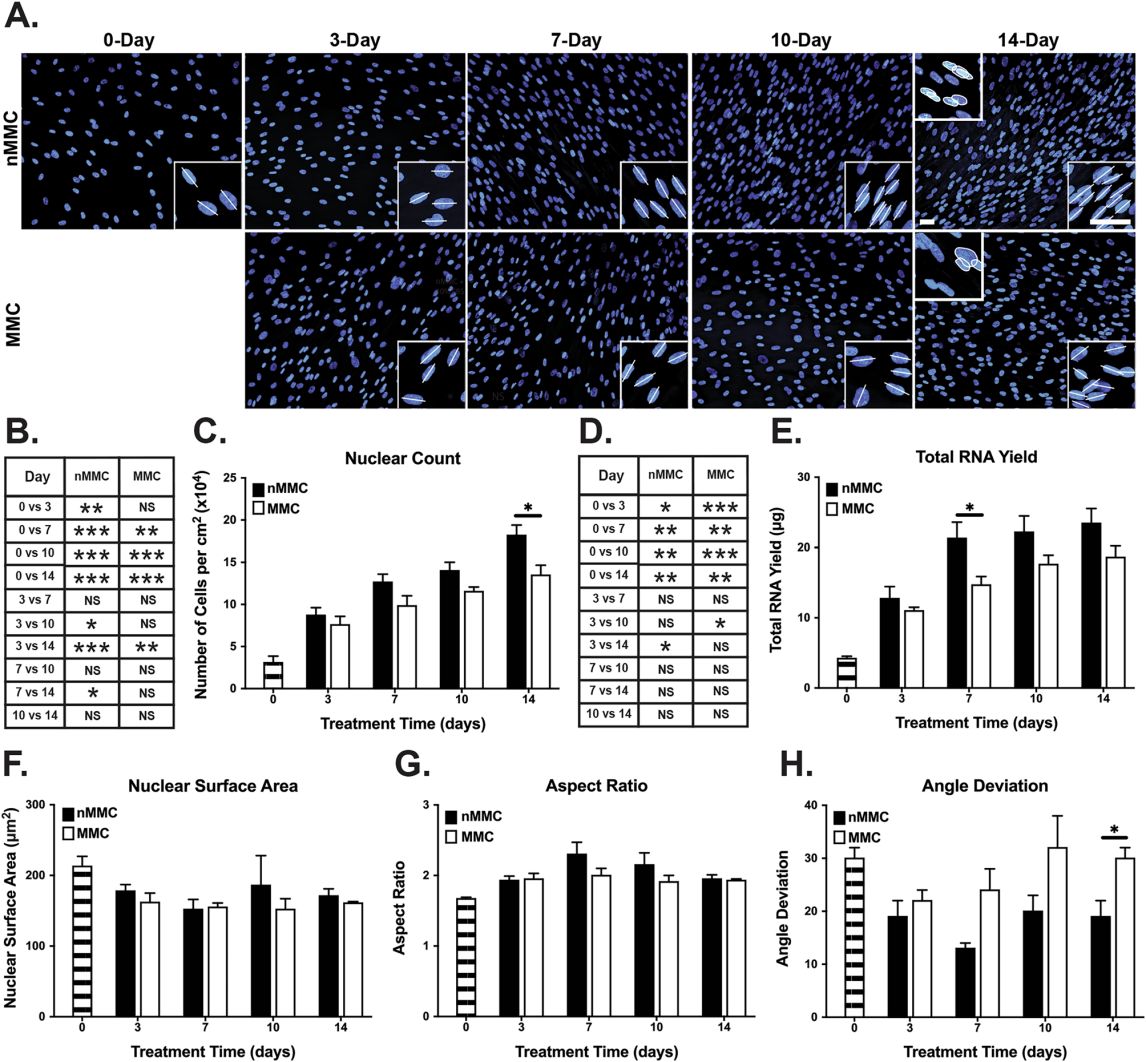
Characterization of cell numbers, morphology, and orientation in MMC and nMMC cultures over time. (**A**) Representative standardized images of the cultures with nuclear DAPI stain over time. Lower inserts show higher magnification images of select areas with long axis of the nuclei indicated by a line. Upper inserts in 14-day images show nuclear overlap as indicated by white tracing of the outlines of nuclei. Magnification bars = 40 μm. (**B**) Statistical comparison of change in cell numbers based on calculation of DAPI stained nuclei over time (n = 4 repeated experiments). (**C**) Pairwise comparison of cell numbers based on calculation of DAPI stained nuclei at each time point (n = 4 repeated experiments). (**D**) Statistical comparison of change in total RNA yield over time (n = 6–7 repeated experiments). (**E**) Pairwise comparison of total RNA yield at each time point (n = 6–7 repeated experiments). Comparison of nuclear surface area (**F**), aspect ratio (**G**), and angle deviation (**H**) in DAPI stained images over time (n = 3 repeated experiments). Results show mean + /− SEM from repeated experiments (**C,E–H**). Statistical testing was performed by one-way ANOVA (**B**,**D**) and independent samples t-test (**C**,**E–H**), *p < 0.05; **p < 0.01; ***p < 0.001.

Phase contrast microscopy (Fig. 1A) and immunostaining of cytoskeletal β-tubulin (Fig. 1B) and actin (Fig. 1C) showed that in both MMC and nMMC culture conditions, cells assumed in general parallel orientation. However, at day 3–14 when cultures had reached confluency, cells in MMC condition appeared to be less densely packed with wider intercellular spaces than in nMMC cultures (Fig. 1A–C). Staining with DAPI showed that, unlike at day 0 (after 24 h culture) where cells formed a monolayer, at day 3 nuclei of some cells overlapped indicating that both MMC and nMMC conditions were composed of layers of GFBLs (data not shown). This nuclear (cell) overlap appeared more common with increasing culture time up to day 14 (Fig. 2A upper inserts). Counting of DAPI-stained nuclei (Fig. 2B) or measurement of total RNA levels (Fig. 2D) showed that in both culture conditions cell numbers increased significantly over time. However, pairwise comparison of nuclear count (Fig. 2C) or RNA yield (Fig. 2E) between the treatments at each time point showed that the number of cells in MMC cultures was significantly lower compared to nMMC condition at days 7 and 14. To assess cell morphology in more detail, we quantified nuclear surface area (Fig. 2F) and aspect ratio (Fig. 2G), measures that correlate with overall cell size and elongation, respectively^28–31^. Pairwise comparison of these measurements in MMC and nMMC cultures did not show any significant differences at any timepoint (Fig. 2F,G, respectively). However, measurement of average angle deviation of the long axis of the nuclei, a measure that describes orientation of long axis of the cells compared to other cells, showed significantly higher angle deviation in MMC compared to nMMC at 14-day cultures (Fig. 2A lower inserts and H). Thus, GFBLs cultured in MMC compared to nMMC display significantly reduced cell numbers over time and more varied orientation compared to nMMC condition.

### MMC modulates gene expression by gingival fibroblasts

Next, we wanted to find out whether MMC regulates gene expression in the GFBL cultures. We focused on genes that encode various ECM proteins or their processing enzymes and genes that associate with ECM remodeling (MMPs and TIMPs), ECM deposition (TGF-β signaling), cell-ECM interactions (integrins), and wound healing (cell–cell communication, myofibroblast differentiation, and angiogenesis). We first assessed expression of a set of 32 genes separately in MMC and nMMC conditions to find out their expression over time (Supplementary Figure S2). Expression of 13 (41%) and 11 (34%) of the above genes was significantly changed over time in MMC and nMMC conditions, respectively (Supplementary Figure S2). Out of these genes in either condition, four were significantly downregulated (*COL4A1, ELN, SPARC and TGFB1*), while 10 were upregulated (*BGN, DCN, FMOD, HAS2, MMP1, MMP10, TGFB2, LTBP2, LTBP3, LTBP4 and SDF1*) over time (Supplementary Figure S2). Majority of the significant gene expression changes in both culture conditions occurred when monolayer cultures (day 0) were compared to the later time points where cultures had formed three-dimensional (3D) layers (Supplementary Figure S2). Expression of all the above genes in both MMC and nMMC conditions had stabilized at day 7 as no significant changes in gene expression were noted after that time point (Supplementary Figure S2).

**Figure 3:**
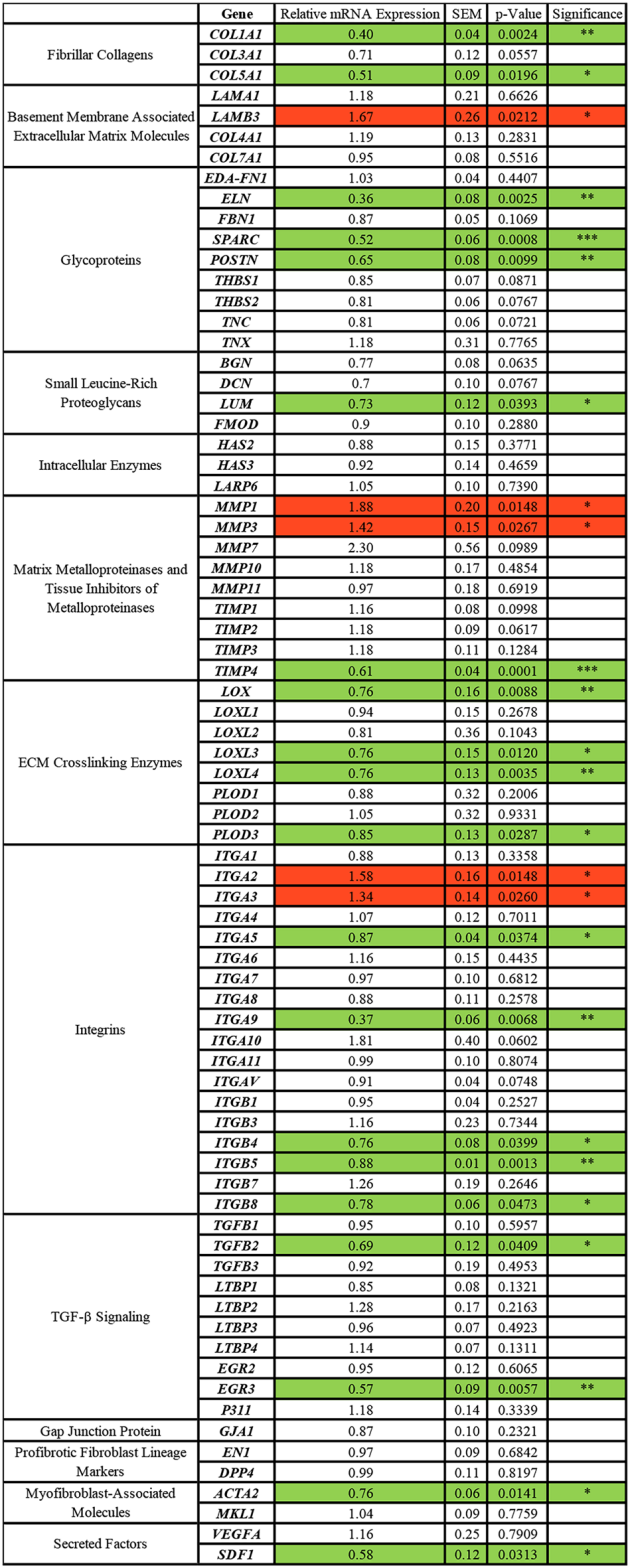
Fibroblast gene expression is modulated by MMC culture condition. Table shows GFBL mRNA expression (mean + /− SEM) of 75 genes in MMC relative to nMMC culture condition after 14 days analyzed by RT-qPCR. Cell color highlights statistically significant downregulation (green) or upregulation (red) of mRNA expression in MMC compared to nMMC culture condition. Statistical testing was performed by independent samples t-test (*p < 0.05; **p < 0.01; ***p < 0.001), n = 4–7 repeated experiments.

Next, we performed pairwise comparisons of gene expression between MMC and nMMC conditions using samples collected from 14-day cultures. Out of the 75 genes analyzed, 25 (33%) were significantly differently expressed in MMC compared to nMMC conditions (Fig. 3). Out of these 25 genes, five were significantly upregulated and 20 downregulated in MMC compared to nMMC cultures (Fig. 3). The upregulated genes encoded for the basement membrane laminin subunit *LAMB3*^32^, laminin receptor integrin α3 subunit (*ITGA3*)^33^, fibrillar collagen receptor integrin α2 subunit (*ITGA2*)^33^, and matrix metalloproteinases *MMP1* and *MMP3* involved in ECM remodeling^34^. In contrast, genes that were downregulated by MMC encoded for several ECM proteins, including collagen I (*COL1A1*), collagen V (*COL5A1*), certain glycoproteins (*ELN, SPARC, POSTN*) and small leucine-rich proteoglycan lumican (*LUM*), *TIMP4* involved in tissue remodeling^35^, *LOX, LOXL3, LOXL4* and *PLOD3* that mediate ECM maturation and cross-linking^36^, various integrin α- and β-subunits, *TGFB2*, *EGR3* involved in TGF-β signaling^37^, *ACTA2* that encodes for α-smooth muscle actin (αSMA), a gene highly expressed by profibrotic myofibroblasts^38^, and *SDF1* (*CXCL12*), a chemokine that regulates angiogenesis^37^ (Fig. 3). Thus, MMC significantly modulates expression of several genes important in establishing and remodeling of GFBL ECM niche.

### MMC promotes accumulation of ECM proteins in gingival fibroblast cultures

**Figure 4:**
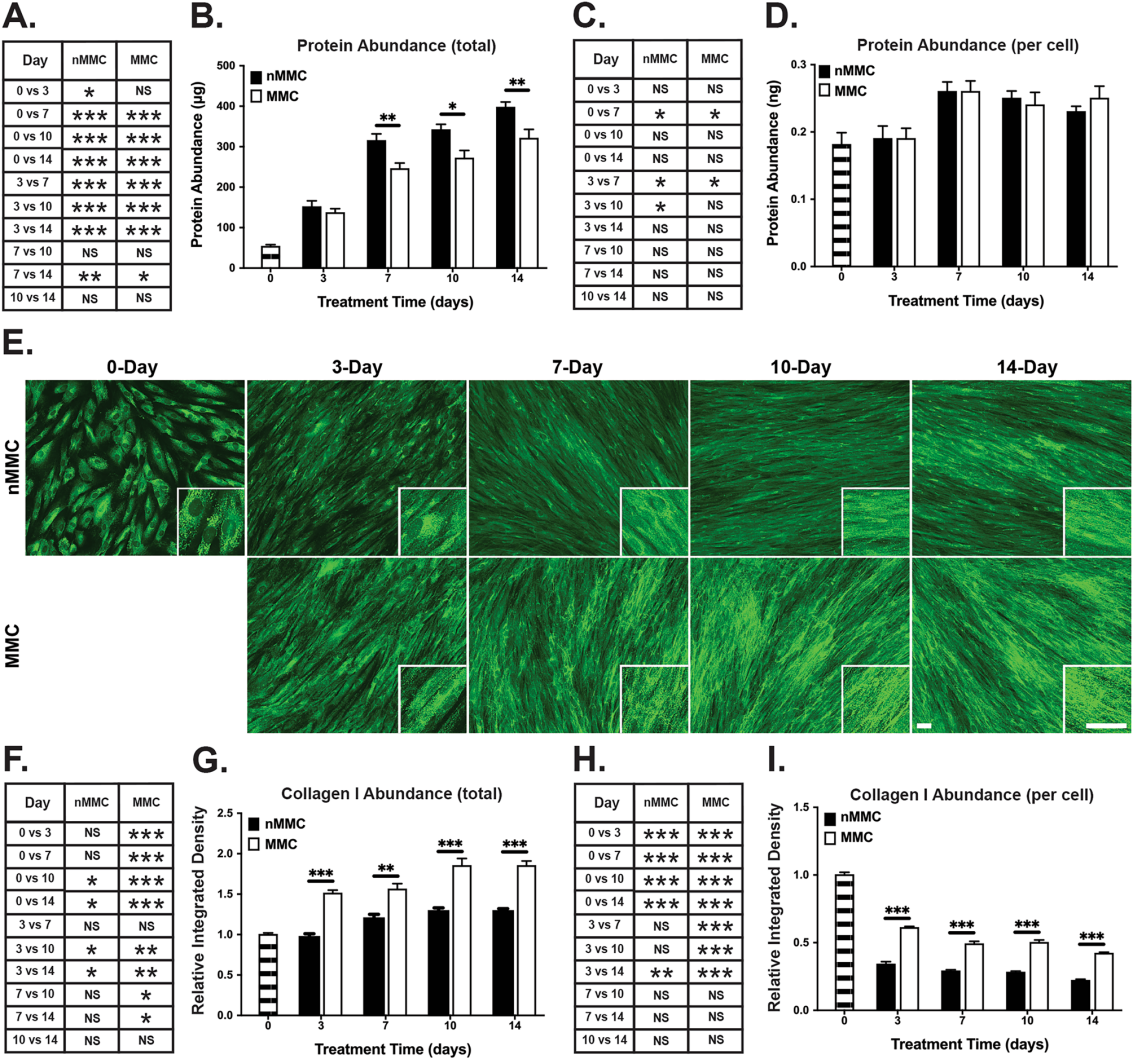
Quantification of total protein amount and characterization of collagen I organization and abundance in MMC and nMMC cultures over time. (**A**) Statistical comparison of change in total protein amount in MMC and nMMC cultures over time. (**B**) Pairwise comparison of total protein abundance at each time point. (**C**) Statistical comparison of change in relative (normalized for cell numbers determined by nuclear DAPI counts) protein abundance over time. (**D**) Pairwise comparison of relative protein abundance at each time point. (**E**) Representative standardized images of collagen I immunostaining from each time point and treatment from two repeated experiments. Inserts show higher magnification of select areas. Images in inserts are individually optimized for brightness/contrast. Magnification bars = 40 μm. (**F**) Statistical comparison of change in total collagen I abundance over time. (**G**) Pairwise comparison of total collagen I abundance at each time point. (**H**) Statistical comparison of change in relative (normalized for cell numbers determined by nuclear DAPI counts) collagen I abundance over time. (**I**) Pairwise comparison of relative collagen I abundance at each time point. Results show mean + /− SEM (**B**,**D**,**G**,**I**) and statistical comparison over time (**A**,**C**,**F**,**H**) from 8 to 11 repeated experiments (**A**–**D**) and from image analysis performed with 6–12 images obtained from each time point and treatment from two repeated experiments (**F**–**I**). Statistical testing was performed by one-way ANOVA (**A**,**C**,**F**,**H**) and by independent samples t-test (**B**,**D**,**G**,**I**), *p < 0.05; **p < 0.01; ***p < 0.001.

In order to find out whether MMC also modulates protein content in the cultures, we quantified total and relative (normalized for cell numbers) protein levels in the cell-ECM layers. Findings showed that total protein levels increased significantly in both culture conditions up to day 14 (Fig. 4A). The most noticeable increase in relative protein levels occurred at day 7 compared to earlier time points in both MMC and nMMC cultures (Fig. 4C). Pairwise comparison showed that MMC cultures had significantly lower levels of total proteins compared to nMMC cultures at days 7–14 (Fig. 4B). However, pairwise comparison of protein amounts relative to cell numbers at each time point showed that there was no significant difference between the two culture conditions (Fig. 4D).

In order to find out whether MMC modulates accumulation of key ECM molecules, we quantified their abundance in the cell-ECM layer in immunostained cultures over time. Collagen I, the major ECM protein produced by fibroblasts^1^, showed strong intracellular immunoreactivity at day 0, but at later time points it gradually displayed predominantly fibrillar extracellular localization in both MMC and nMMC cultures (Fig. 4E). In MMC condition, total collagen I increased significantly up to day 10, while there was no significant change after day 7 in nMMC cultures (Fig. 4F,G). When collagen I abundance was assessed relative to cell numbers, it showed significantly decreasing staining intensity over time in both MMC and nMMC conditions (Fig. 4H,I). Pairwise comparison of total (Fig. 4G) and relative (Fig. 4I) collagen I in MMC and nMMC cultures showed strongly increased levels in MMC cultures at day 3–14.

**Figure 5:**
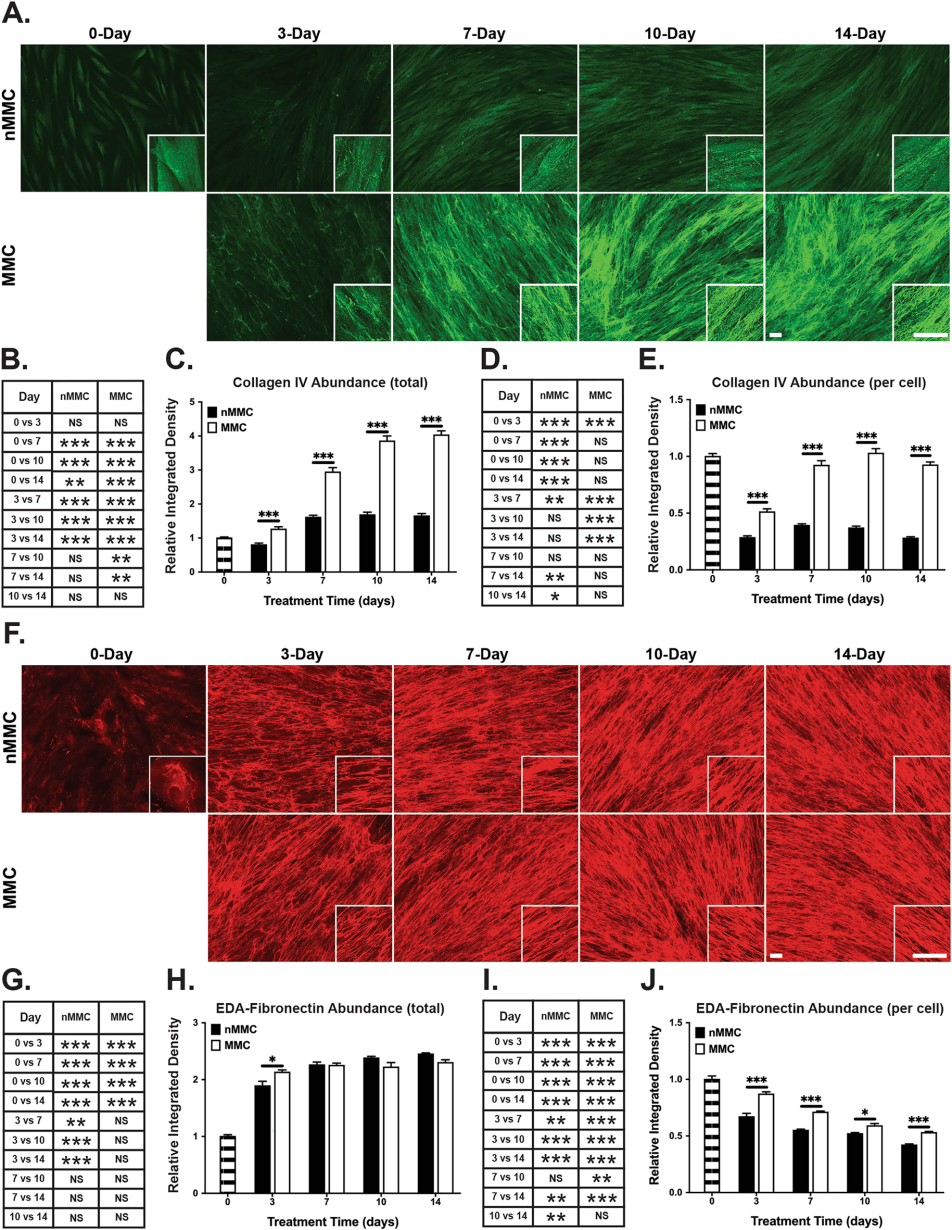
Characterization of collagen IV and cellular fibronectin organization and abundance in MMC and nMMC cultures over time. (**A**) Representative standardized images of collagen IV immunostaining from each time point and treatment from two repeated experiments. Inserts show higher magnification of select areas. Images in inserts are individually optimized for brightness/contrast. (**B**) Statistical comparison of change in total collagen IV abundance over time. (**C**) Pairwise comparison of total collagen IV abundance at each time point. (**D**) Statistical comparison of change in relative (normalized for cell numbers determined by nuclear DAPI counts) collagen IV abundance over time. (**E**) Pairwise comparison of relative collagen IV abundance at each time point. (**F**) Representative standardized images of cellular fibronectin (EDA-fibronectin) immunostaining from each time point and treatment from two repeated experiments. Inserts show higher magnification of select areas. Images in inserts are individually optimized for brightness/contrast. (**G**) Statistical comparison of change in total cellular fibronectin abundance over time. (**H**) Pairwise comparison of total cellular fibronectin abundance at each time point. (**I**) Statistical comparison of change in relative (normalized for cell numbers determined by nuclear DAPI counts) cellular fibronectin abundance over time. (**J**) Pairwise comparison of relative cellular fibronectin abundance at each time point. Results show mean + /− SEM (**C**,**E**,**H**,**J**) and statistical comparison over time (**B**,**D**,**G**,**I**) from image analysis performed with 6–12 images obtained from each time point and treatment from two repeated experiments. Statistical testing was performed by one-way ANOVA (**B**,**D**,**G**,**I**) and by independent samples t-test (**C**,**E**,**H**,**J**), *p < 0.05; **p < 0.01; ***p < 0.001. Magnification bars = 40 μm (**A**,**F**).

Similar to collagen I, collagen IV, a basement membrane molecule^32^, also localized mostly intracellularly at day 0, but at the later timepoints it showed increasingly extracellular fibrillar accumulation (Fig. 5A). In MMC cultures, abundance of total collagen IV increased significantly up to 10 days, while there was no significant change after day 7 in nMMC cultures (Fig. 5B,C). When collagen IV levels were compared relative to cell numbers over time, there was a significantly decrease in nMMC condition at all time points compared to day 0 (Fig. 5D,E). In contrast, MMC cultures showed a significant decrease only at day 3, after which collagen IV levels reached similar relative level as found at day 0 (Fig. 5D,E). Similar to collagen I, pairwise comparison of collagen IV at each time point showed strongly increased total (Fig. 5C) and relative (Fig. 5E) levels in MMC compared to nMMC cultures at all time points.

Immunostaining for cellular fibronectin (EDA-fibronectin), a key cell adhesion ligand and molecule involved in ECM assembly^1^, was also mostly localized intracellularly at day 0, but both culture conditions showed strong fibrillar extracellular fibronectin staining at the later time points (Fig. 5F). In both cultures, abundance of total fibronectin increased strongly over time compared to day 0, and reached peak levels by day 3 and 7 in MMC and nMMC cultures, respectively (Fig. 5G,H). When fibronectin levels were compared relative to cell numbers over time, there was a significant progressive decrease in both MMC and nMMC cultures at all time points (Fig. 5I,J). Pairwise comparison of total (Fig. 5H) fibronectin levels at each time point showed only minor differences between MMC and nMMC cultures. However, relative fibronectin levels in MMC cultures were significantly higher compared to nMMC cultures at all time points (Fig. 5J).

**Figure 6:**
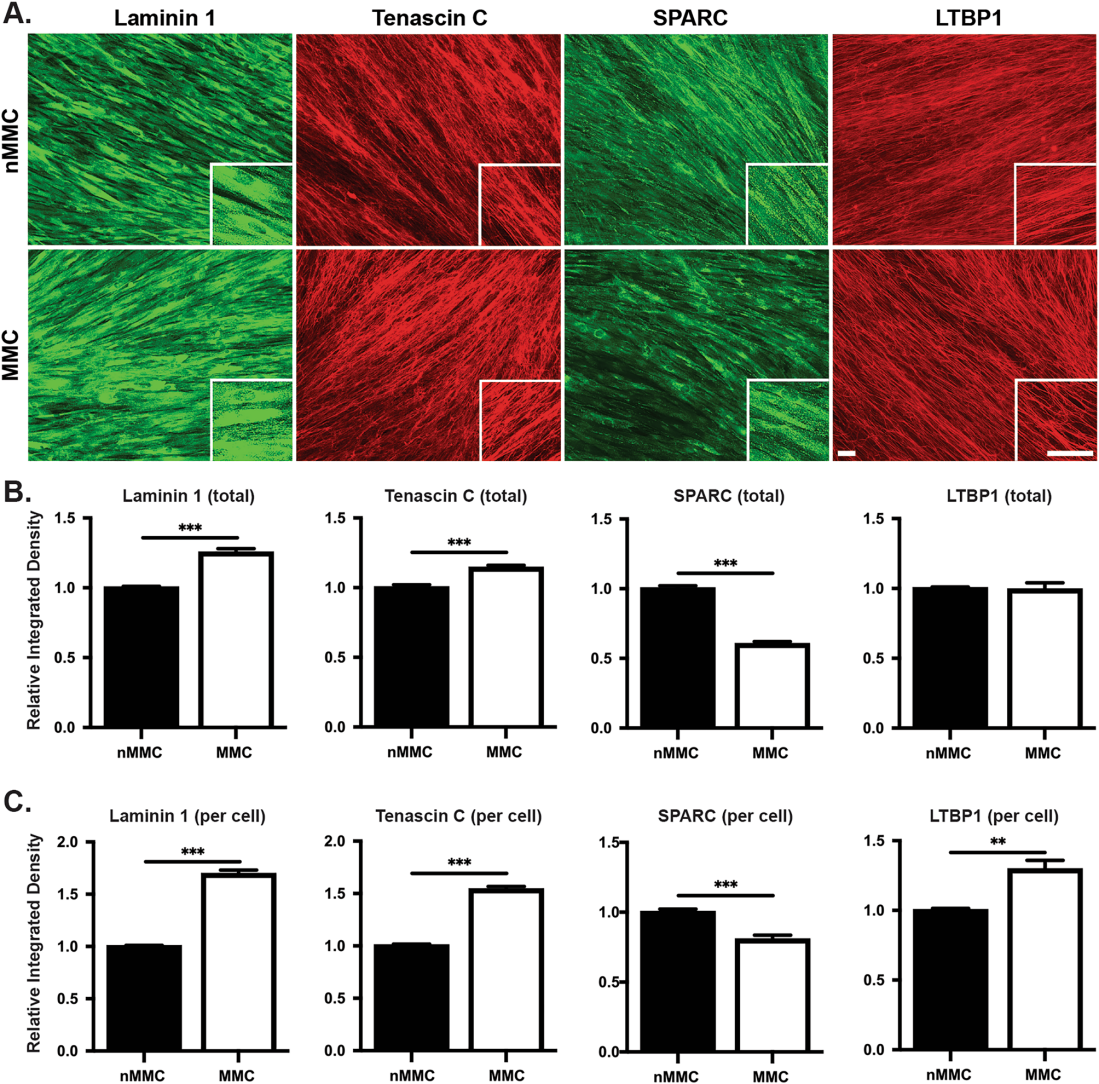
Characterization of laminin 1, tenascin C, SPARC, and LTBP1 organization and abundance in MMC and nMMC cultures at day 14. (**A**) Representative standardized images of laminin 1, tenascin C, SPARC, and LTBP1 immunostaining from each time point and treatment from two repeated experiments. Inserts show higher magnification of select areas. Images in inserts are individually optimized for brightness/contrast. Magnification bars = 40 μm. Pairwise comparison of total (**B**) and relative (normalized for cell numbers determined by nuclear DAPI counts) (**C**) abundance of laminin 1, tenascin C, SPARC, and LTBP1 at day 14. Results show mean + /− SEM from image analysis performed with 6–12 images obtained from each time point and treatment from two repeated experiments (**B**,**C**). Statistical testing was performed by independent samples t-test, **p < 0.01; ***p < 0.001.

We then analyzed abundance of the basement membrane component laminin 1^32^, tenascin C (TNC) and SPARC (osteonectin), two matricellular glycoproteins that modulate ECM accumulation and cell function in wound healing^39,40^, and latent TGF-β-binding protein 1 (LTBP1), a protein that binds and stores profibrotic latent TGF-β into the ECM^41^, in day 14 cultures (Fig. 6A–C). Staining for laminin 1 and SPARC appeared mostly intracellular, while tenascin C and LTBP1 showed a fibrillar, ECM-associated staining pattern in both culture conditions (Fig. 6A). Quantitation of immunostaining showed significantly higher levels of total (Fig. 6B) and relative (Fig. 6C) laminin 1 and tenascin C and relative level of LTBP1 (Fig. 6C) in MMC compared to nMMC conditions. In contrast, MMC cultures contained significantly lower total and relative levels of SPARC compared to nMMC condition (Fig. 6B,C). Taken together, compared to nMMC condition, MMC promotes significantly accumulation of collagen I and IV, cellular fibronectin, laminin 1, tenascin C and LTBP1, while reducing abundance of SPARC in the cell-ECM layer of GFBL cultures.

### MMC suppresses expression of myofibroblast markers in gingival fibroblasts

**Figure 7:**
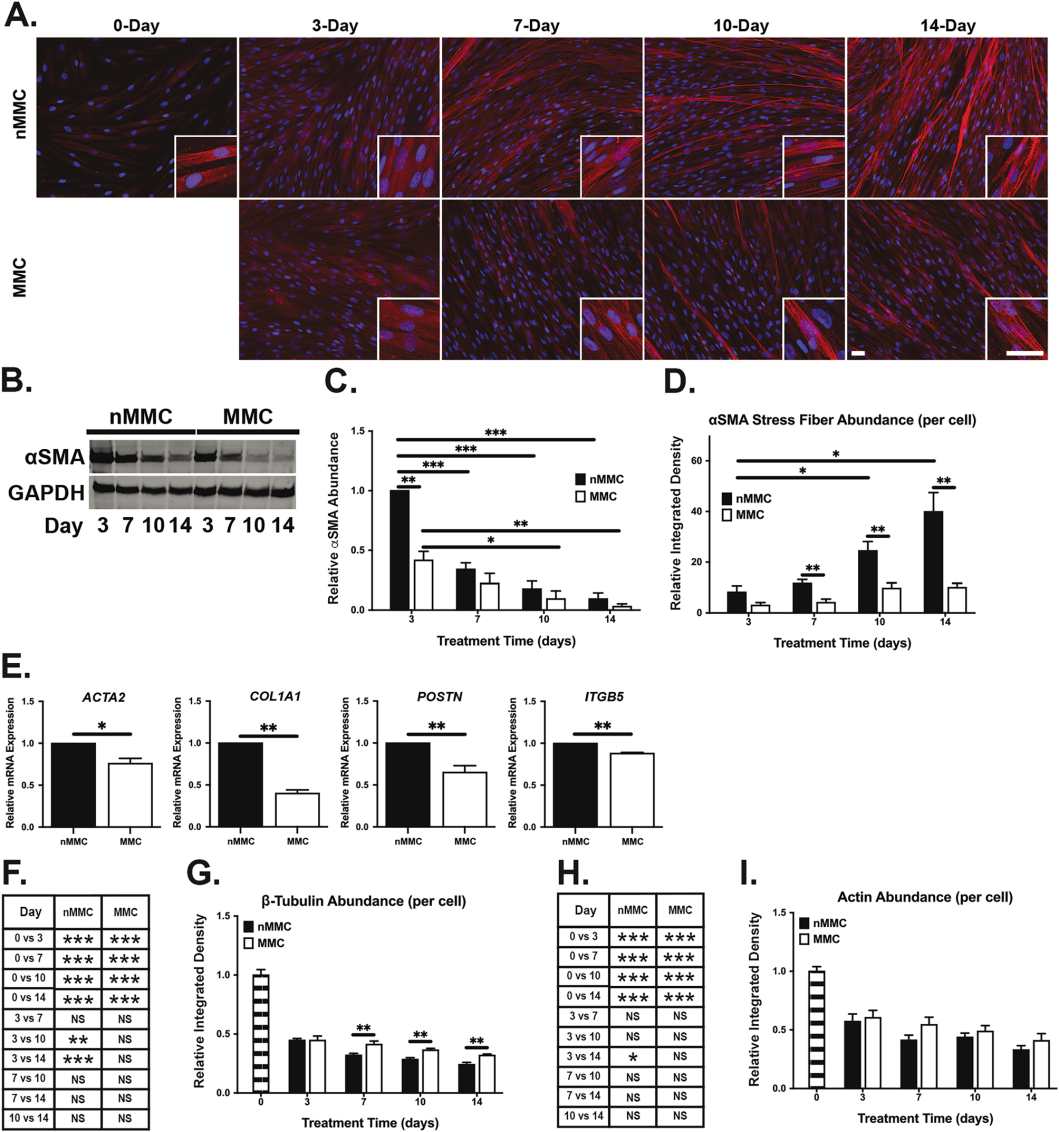
Characterization of myofibroblast-associated markers and cytoskeletal proteins in MMC and nMMC cultures. (**A**) Representative standardized images of αSMA immunostaining from each time point and treatment from two repeated experiments. Inserts show higher magnification of select areas. Images in inserts are individually optimized for brightness/contrast. Magnification bars = 40 μm. (**B**) Representative Western blot of αSMA in MMC and nMMC cultures at day 3–14. GAPDH was used as loading control. Cropped images of Western blots are shown. (**C**) Quantitation and statistical comparison of change in αSMA abundance at each time point and over time relative to GAPDH determined by Western blotting. Results show mean + /− SEM from four repeated experiments. (**D**) Quantification (mean + /− SEM) of αSMA stress fibers by image analysis over time and in pairwise comparison at each time point. (**E**) Quantification (mean + /− SEM) of expression of *ACTA2*, *COL1A1*, *POSTN* and *ITGA5* by RT-qPCR in MMC relative to nMMC cultures at day 14 (n = 4–7 repeated experiments). (**F**) Statistical comparison of change in relative (normalized for cell numbers determined by nuclear DAPI counts) β-tubulin abundance over time. (**G**) Pairwise comparison of relative β-tubulin abundance at each time point. (**H**) Statistical comparison of change in relative (normalized for cell numbers determined by nuclear DAPI counts) actin abundance over time. (**I**) Pairwise comparison of relative actin abundance at each time point. Results show mean + /− SEM (**D**,**G**,**I**) and statistical comparison over time (**F**,**H**) from image analysis from 6–12 images obtained from each time point and treatment from two repeated experiments. Statistical comparison between time points was performed by one-way ANOVA (**C**,**D**,**F**,**H**) and between treatments at each time point by independent samples t-test (**C**,**D**,**E**,**G**,**I**), *p < 0.05; **p < 0.01; ***p < 0.001.

ECM regulates fibroblast differentiation into profibrotic myofibroblasts in vitro and in vivo. Characteristics of myofibroblasts include high expression of *ACTA2*, a gene that encodes for αSMA^41^. Therefore, we next studied levels of αSMA in the two culture conditions over time (Fig. 7A–D). Based on Western blotting (Fig. 7B,C) relative αSMA abundance decreased significantly in both cultures over time. Pairwise comparison at each time point showed that αSMA levels were significantly lower in MMC compared to nMMC condition at day 3 (Fig. 7C). Next, we assessed organization of αSMA into stress fibers, one of the characteristics associated with differentiation of fibroblasts into myofibroblasts^41^. At day 0, αSMA showed a diffuse cytoplasmic staining pattern and was not organized into stress fibers (Fig. 7A). Some cells with αSMA positive stress fibers started to appear in both MMC and nMMC cultures at day 3, which became more prevalent in nMMC but not in MMC cultures over time (Fig. 7A). Accordingly, quantification of αSMA stress fibers relative to cell numbers showed a significant increase in nMMC cultures while there was no change in MMC cultures over time (Fig. 7D). Pairwise comparison showed significantly lower levels of αSMA stress fibers in MMC compared to nMMC cultures at day 7–14 (Fig. 7D). To assess whether other features associated with myofibroblast differentiation are also regulated in MMC compared to nMMC condition, we reviewed myofibroblast related gene expression in the 14-day cultures. Similar to significantly higher expression of *ACTA2* (αSMA) mRNA by GFBLs cultured in nMMC compared to MMC condition, they also expressed significantly higher levels of *COL1A1*, *POSTN* and *ITGB5* (Figs. 3 and 7E), genes previously shown to be highly expressed by myofibroblasts^41^. Analysis of other cytoskeletal proteins showed significantly higher relative β-tubulin but similar actin levels in MMC compared to nMMC cultures (Fig. 7F–I). In summary, MMC condition specifically suppresses GFBL αSMA levels compared to other cytoskeletal proteins and reduces levels of myofibroblast differentiation markers compared to nMMC cultures over time.

### Novel decellularization method to generate cell-free cell-derived matrices

**Figure 8:**
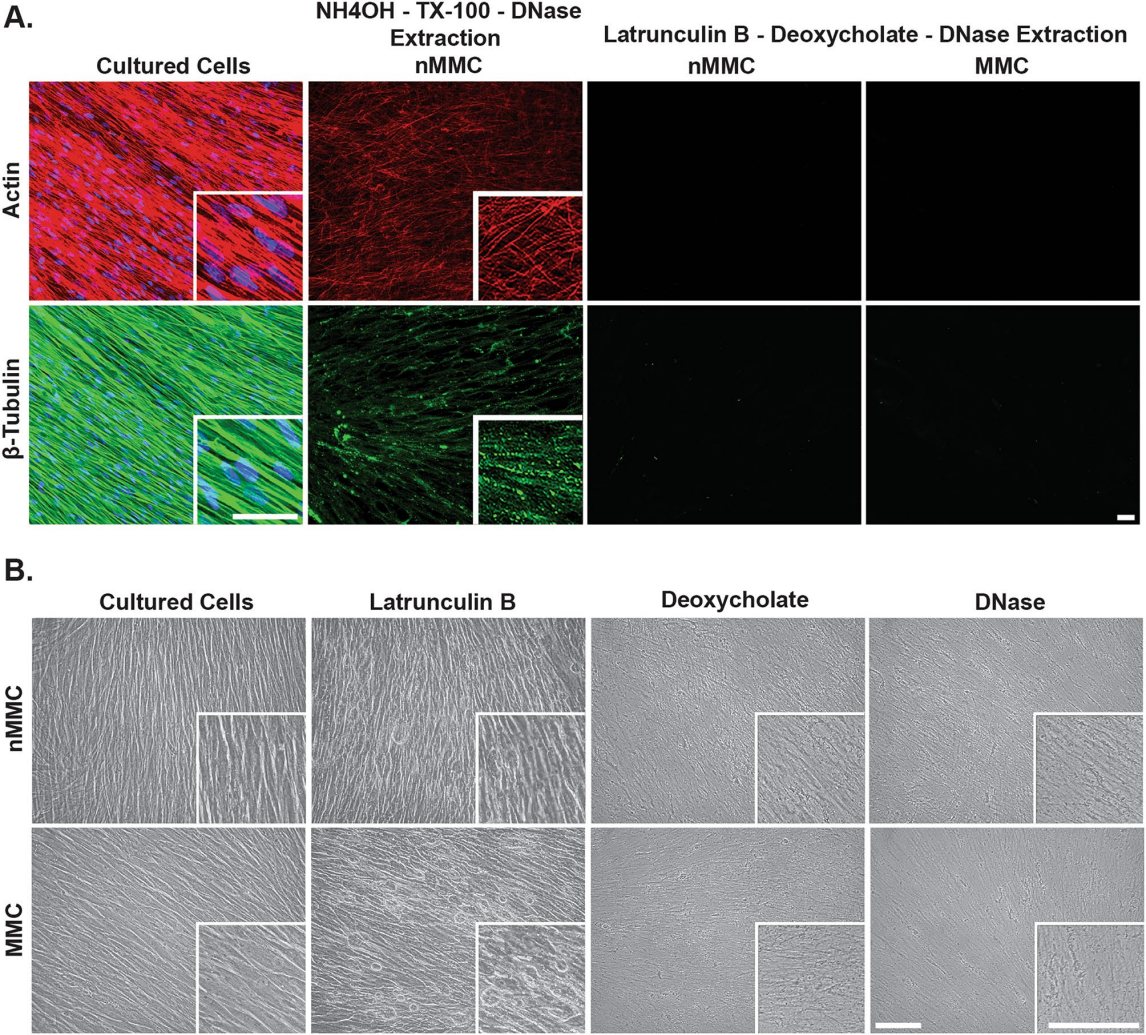
Characterization of decellularization methods. (**A**) Representative standardized immunofluorescence images of actin and β-tubulin in 14-day MMC and nMMC cultures before and after NH_4_OH-TX-100-DNase or latrunculin B-deoxycholate-DNase decellularization protocols. DNA was stained with DAPI nuclear stain (blue). (**B**) Representative standardized phase contrast microscope images of 14-day MMC and nMMC cultures before and after sequential incubations with latrunculin B, deoxycholate, and DNase. Inserts show higher magnification of select areas. Images in inserts are individually optimized for brightness/contrast. Magnification bars = 40 μm.

Decellularization can be used to generate cell-free cell-derived matrices (CDMs) from 3D fibroblast cultures. A widely used decellularization method involves use of an alkaline NH_4_OH (20 mM)/Triton X-100 (0.5%) and DNase extraction protocol^42^. However, our findings from 14-day nMMC cultures of GFBLs indicated that this treatment does not completely remove cytoskeletal actin and β-tubulin from the cultures (Fig. 8A). Therefore, in order to compare CDMs generated in presence or absence of MMC, we first tested efficiency of a novel decellularization method using treatment with 0.6 μM latrunculin B for 3 h to destabilize the actin cytoskeleton followed by incubations with 0.5% sodium deoxycholate and DNase. Phase contrast microscopy of 14-day MMC and nMMC cultures showed that latrunculin B treatment caused rounding of cells, which appeared more pronounced in the MMC than in the nMMC cultures (Fig. 8B). Subsequent treatments with sodium deoxycholate and DNase resulted in removal of all notable cellular elements from both cultures leaving behind a fibrillar ECM (Fig. 8B). Immunostaining of actin and β-tubulin, and of DNA by DAPI, confirmed efficient removal of these elements from the cultures (Fig. 8A).

### Comparison of composition of gingival fibroblast CDMs generated with or without MMC

**Figure 9:**
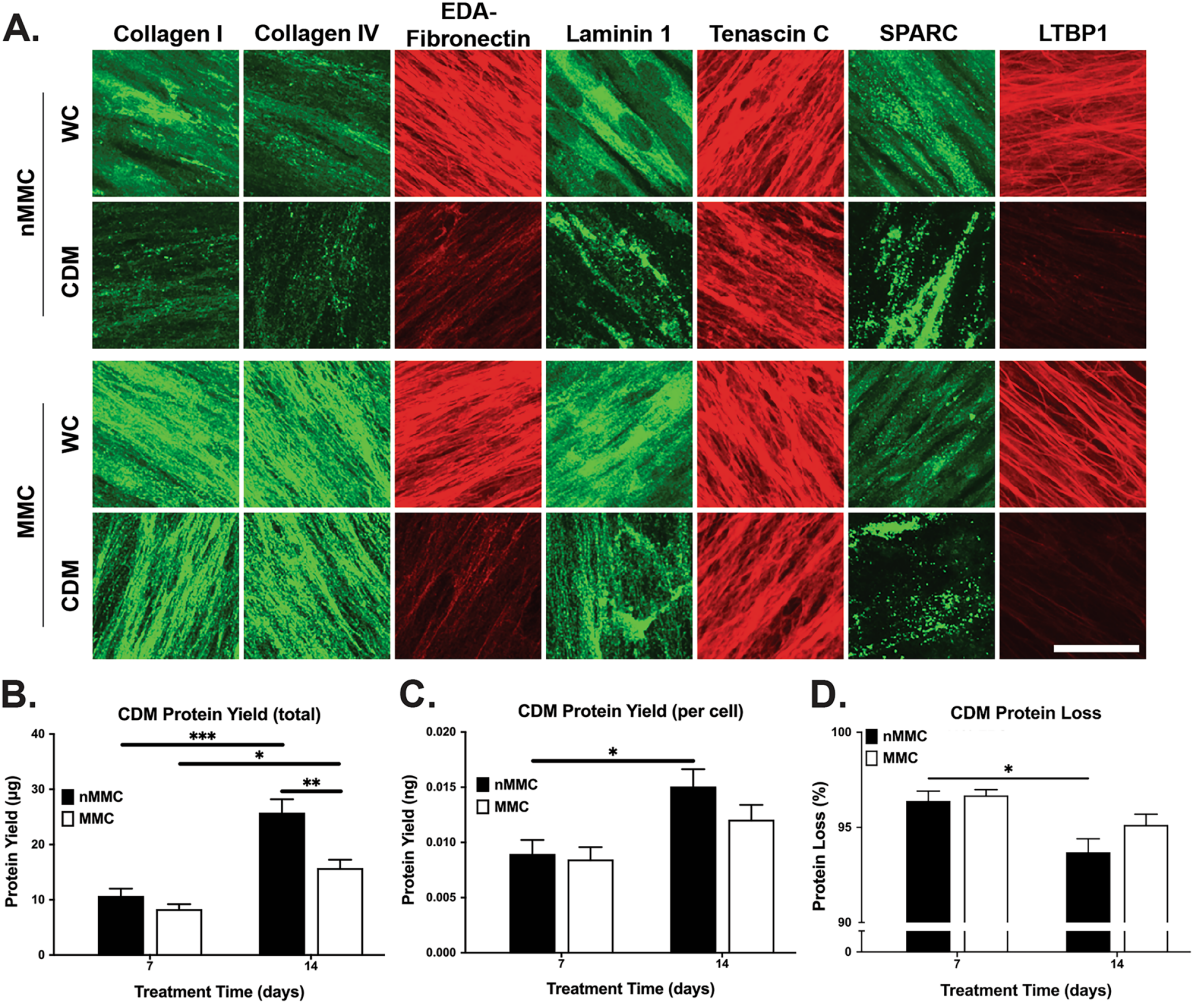
Characterization of cultures and CDMs generated in MMC and nMMC conditions before and after decellularization. (**A**) Representative standardized images of collagen I, collagen IV, cellular fibronectin (EDA-fibronectin), laminin 1, tenascin C, SPARC, and LTBP1 immunostaining in 14-day MMC and nMMC cultures with cells (WC) and after decellularization (CDM) from two repeated experiments. Magnification bar = 40 μm. (**B**,**C**) Comparison of total (**B**) and relative (normalized for cell numbers based on nuclear DAPI counts in parallel samples) (**C**) protein amount over time and between MMC and nMMC CDMs. (**D**) Comparison of total protein loss due to decellularization over time between MMC and nMMC CDMs. Results show total protein amount (mean + /− SEM) from CDMs relative to corresponding cultures with cells (= 100%) as determined by Bradford assay. (**B**–**D**) Results show mean + /− SEM from 6 repeated experiments. Statistical testing between time points was performed by one-way ANOVA and between treatments at each time point by independent samples t-test, *p < 0.05; **p < 0.01; ***p < 0.001.

Next, to compare CDMs generated using the above novel decellularization protocol, we quantified the total proteins in CDMs from 7- and 14-day MMC and nMMC cultures. Similar to cultures containing cells (Fig. 4A), in both MMC and nMMC CDMs, total protein amounts increased significantly from day 7 to 14 (Fig. 9B). Relative protein levels increased over time as well, but only measurements from nMMC cultures reached statistical significance (Fig. 9C). Pairwise comparison showed that CDMs from 14-day nMMC cultures had accumulated significantly higher amounts of total proteins compared to corresponding MMC cultures (Fig. 9B). However, similar to the above findings from cultures containing cells (Fig. 4D), when the protein amounts in the CDMs were normalized for cell numbers (Fig. 9C), there were no significant differences between the two culture conditions. We then studied the above ECM molecules in immunostained CDMs from day 3–14 cultures. The findings showed that the differences noted in total proteins and a set of ECM proteins between cultures generated with MMC compared to nMMC were replicated in the cell-free CDMs generated from the corresponding cultures after decellularization (Supplementary Figures S3–5).

### Characterization of protein loss due to decellularization in gingival fibroblast CDMs generated with or without MMC

There was a notable decrease of immunostaining intensity of the ECM molecules in CDMs compared to GFBLs-containing cultures (Fig. 9A) indicating that decellularization process caused this loss. To quantify protein loss in more detail, we first compared abundance of total proteins (Fig. 9B) in parallel GFBLs-containing cultures and CDMs. On average total protein yield from MMC cultures at day 7 and 14 was 240 μg/9.6 cm^2^ and 320 μg/9.6 cm^2^ culture well, respectively. After decellularization, the corresponding protein yields were reduced to 8 μg and 16 μg, respectively. For nMMC cultures the values were 314 μg/9.6 cm^2^ and 397 μg/9.6 cm^2^ before decellularization, and 11 μg and 26 μg after decellularization, respectively. Pairwise comparison of MMC and nMMC cultures indicated that there was no significant difference in protein loss between the two conditions (Fig. 9D). However, pairwise comparison at each timepoint showed that cultures generated with MMC lost significantly less collagen I compared to nMMC cultures, ranging from 42% to 13% in MMC and 55% to 30% in nMMC CDMs at 3 to 14-day time points, respectively (Supplementary Figure S6). In contrast, both MMC and nMMC cultures displayed in general similar collagen IV and fibronectin loss. Pairwise comparison of tenascin C (loss of about 19%), SPARC (about 53%) and LTBP1 (about 75%), which were assessed in 14-day cultures, showed also protein loss after decellularization that was quantitatively similar in both MMC and nMMC cultures (Supplementary Figure S6). In contrast, loss of laminin 1 was significantly lower in MMC (about 57%) compared to nMMC cultures (about 66%) (Supplementary Figure S6). Taken together, compared to nMMC condition, MMC improved significantly retention of collagen I and laminin 1 in the cultures after decellularization. However, decellularization caused marked loss of total proteins and the studied individual ECM molecules in both MMC and nMMC cultures.

## Discussion

The purpose of MMC is to generate conditions where molecules are in a crowded environment similar to tissues. Our findings showed that MMC promoted deposition of several ECM molecules in the cell-ECM layer in the gingival fibroblast cultures. In particular, accumulation of key ECM proteins (collagen I, cellular fibronectin, tenascin C, and LTBP1) and basement membrane components (collagen IV and laminin 1) were significantly increased by MMC. In contrast, abundance of SPARC, a matricellular protein that modulates cell-ECM interactions in wound healing^40^, was significantly reduced by MMC. Thus, effect of MMC on accumulation of different ECM proteins appears distinct. In general, previous studies using primary human fibroblasts from different tissues, including cornea or different areas of skin, have also demonstrated the ability of MMC to promote accumulation of various ECM proteins, including collagen I, III, IV, V, and VII, fibronectin, perlecan, and laminin 1. However, results have been variable possibly due to differences in culture conditions, MMCs used, and origin of cells^9,43–48^.

The above data about distinct abundance of different ECM molecules in MMC cultures suggests that mechanical properties of the ECM-niche may also be different compared to nMMC cultures. In fact, MMC has been shown to reduce stiffness of ECM by about 60–70% in cultures of neonatal fibroblasts^47,48^. Mechanosignalling from ECM is a powerful regulator of gene expression^49^. Therefore, according to principle of dynamic reciprocity^1,50–53^, MMC may promote deposition of compositionally and mechanically distinct ECM-niche by fibroblasts, which in turn may regulate various fibroblasts functions such as proliferation, morphology, orientation, and gene expression. In support of this, our findings also showed that MMC significantly changed the expression of 25 out of 75 wound healing and ECM-related genes compared to nMMC condition at the 14-day endpoint. In particular, MMC induced significant downregulation of 20 genes that encoded six ECM proteins (*COL1A1*, *COL5A1*, *ELN*, *SPARC*, *POSTN*, and *LUM*), *TIMP4*, *LOX, LOXL3, LOXL4 and PLOD3* that modulate ECM maturation and stability^35,36^, various integrin α- and β-subunits involved in cell-ECM interactions, TGF-β signaling molecules (*TGFB2* and *EGR3*) that among other things promote ECM deposition by fibroblasts^54,55^, myofibroblast-associated *ACTA2*^41^, and proangiogenic *SDF1*^37^. In contrast, MMC upregulated expression of *MMP1* and *MMP3*, two enzymes involved in ECM remodeling^56,57^, *ITGA2* and *ITGA3,* genes encoding integrin subunits involved in ECM remodeling and cell adhesion to collagen and laminin, respectively^56,57^, and *LAMB3* that encodes the β-chain present in laminin 5 (LM 332)^58^. Thus, MMC suppressed expression of a set of genes that are involved in ECM deposition and increased those involved in ECM remodeling in gingival fibroblasts, suggesting an anti-fibrotic effect. Interestingly, when we compared gene expression over time, no significant expression changes were noted after day 7, suggesting that while cultures still evolved with regards to protein accumulation and cell numbers, the key conditions that regulate gene expression in both MMC and nMMC cultures were already established during the first 7 days. While extensive gene expression analyses as performed in this study have not been reported previously, altered expression of a set of genes, including increased expression and enzymatic activity of MMPs has been also found in human dermal fibroblast cultures treated with MMC (Ficoll 70/400)^46,48^. However, MMC (Ficoll 70/400) did not alter expression of various collagens, fibronectin, and certain cell markers including αSMA in human corneal fibroblasts^43^. Thus, gene expression response to MMC treatment may be cell type-specific.

Our results showing reduced cell numbers and apparent wider intercellular spaces between cells in MMC cultures suggests that the density of cells in MMC is lower compared to nMMC cultures. The mechanisms by which MMC affects cell numbers are not known in detail. It is possible that the ECM niche established under MMC is less conducive for cell proliferation and/or promotes quiescence, apoptosis, necrosis or senescence resulting in a proportional change in proliferating and non-proliferating cells. These processes can be modulated by various factors, including specific interactions of cell surface receptors with distinct ECM ligands, mechanical signals from ECM, and activation of ECM-bound growth factors^50,59–62^ that maybe distinct under MMC culture condition. In addition, the transcriptomic change induced by MMC may be also related to reduced cell numbers and/or density of the cultures. This is supported by previous studies with 3T3 and primary fibroblasts showing that cell growth, and/or increased cell–cell contacts due to higher cell density, can be linked to regulation of gene expression or protein levels^63–71^. In addition, altered cell morphology, that may also occur in response to higher cell density, can result in distinct gene expression^72^. We also noted significantly more variable orientation of the long axis of the cells and apparently wider cell morphology and intercellular spacing based on immunostaining of filamentous β-tubulin cytoskeleton in MMC compared to nMMC cultures. Thus, the topological and other cues from the ECM, which define cell orientation, morphology and function^73^, may also be modulated by MMC.

Increased accumulation of total proteins both in MMC and nMMC cultures over time can be explained by increasing cell numbers in both culture conditions. It is noteworthy, however, that similar to primary human dermal fibroblasts cultured with MMCs (Ficoll 70/400)^44^, gingival fibroblasts showed significantly reduced cell numbers in MMC compared to nMMC cultures. Consequently, while total protein accumulation in the cell-ECM layers was significantly higher in nMMC cultures, when results were normalized for cell numbers, both MMC and nMMC cultures showed similar protein levels. Comparison of MMC-induced gene expression and accumulation of corresponding ECM proteins in the cell-ECM layer showed different results. For instance, while quantitation of collagen I and IV, cellular fibronectin, laminin 1, tenascin C, and LTBP1 showed increased protein accumulation in MMC cultures, expression of these genes was downregulated (*COL1A1*) or unchanged (*COL4A1*, *EDA-FN1*, *TNC*, *LAMA1*, and *LTBP1*) by MMC. ECM proteins, including collagen, produced by fibroblasts can be secreted both into the culture medium and assembled into the cell-ECM layer. Use of MMC is expected to promote accumulation of secreted proteins into the cell-ECM layer. Therefore, increased accumulation of the above molecules is likely caused by effect of MMC to promote their deposition from the medium to the cell-ECM layer rather than MMC-induced transcriptional regulation of gene expression. The only exception for the above was SPARC that showed reduced abundance in MMC cultures at both protein and mRNA levels. Thus, SPARC levels may be regulated by transcriptional mechanisms that suppress its expression under the MMC culture condition. Another possibility is that remodeling of ECM, including collagen, by changes in MMP/TIMP ratio, or its maturation that affects its stability, maybe altered. However, the gene expression analysis showed that while MMC significantly upregulated *MMP1* and *MMP3*, it downregulated expression of MMP inhibitor *TIMP4* and *LOX, LOXL3, LOXL4* and *PLOD3,* which mediate ECM crosslinking. Thus, it appears that increased ECM stability may not explain increased ECM accumulation by MMC, albeit measurement of mRNA levels do not necessarily reflect protein abundance or enzymatic activity.

Interestingly, MMC also potently downregulated markers of profibrotic myofibroblast differentiation. Studies have indicated that fibroblast cultures in serum containing medium resemble granulation tissue in early wound healing where cells are actively proliferating and depositing ECM. In addition, fibroblasts in such culture condition express a transcriptome that resembles early wound healing^71,74^. Therefore, it is interesting that MMC may drive gingival fibroblasts in this wound-like situation to a less profibrotic phenotype. Similar suppression of αSMA levels suggesting reduced myofibroblast differentiation have also been reported with cultures of primary human dermal fibroblasts using MMC (Ficoll 70/400)^44^. Considering the above changes of αSMA stress fibers over time, it was interesting to note that in both nMMC and MMC cultures total αSMA levels (stress fiber-associated and non-associated αSMA) were significantly reduced with increasing culture time. Thus, it appears that considerably higher proportion of αSMA in nMMC cultures is assembled into stress fibers compared MMC cultures. Expression of myofibroblast markers, including αSMA, are regulated by various factors, such as composition of ECM, including abundance of cellular fibronectin, mechanosignalling from ECM, cell density, and presence of growth factors such as TGF-β1^41,75^, but their role in MMC-mediated suppression of myofibroblast phenotype is not known.

MMC also promoted deposition/expression of molecules that are associated with the basement membrane. In particular, abundance of type IV collagen and laminin 1 was significantly increased by MMC. In addition, expression of mRNA for *LAMB3*, was significantly increased, while expression of *COL7A1*, encoding collagen VII that forms the anchoring fibrils^9^, was unaltered by MMC. Previous findings using primary abdominal human skin fibroblasts have demonstrated increased deposition of collagen IV and VII by treatment with MMCs (Ficoll 70/400)^9^, while studies using neonatal human skin fibroblasts have shown conflicting results whether MMC (Ficoll 70/400) promotes laminin 1 deposition^48,76^. Thus, the property of MMC to promote deposition/expression of basement membrane zone molecules by fibroblasts may depend on phenotype and/or origin of fibroblasts. ECM niche present at the basement membrane zone is a critical regulator of growth and function of epithelial stem cells^77^. Quiescent fibroblasts that reside in normal connective tissue do not appear to actively secrete basement membrane molecules such as collagen IV and laminin 1 and 5. However, upon activation during certain physiological or pathological processes, such as development, wound healing, and cancer stroma or in organotypic cultures with keratinocytes, they participate in deposition of these molecules^78–88^. Although published data is scarce, fibroblasts produce only limited amounts basement membrane molecules in standard cultures and this maybe also cell type-specific. For instance, compared to human gingival fibroblasts, skin fibroblasts appear to produce only negligible levels of laminin 5 (LM 332) in culture^89^. The ability of MMC to promote deposition of basement membrane components by gingival fibroblasts may open a possibility to generate improved organotypic cultures consisting of epithelial cells and fibroblasts to study epithelial-mesenchymal interactions and to be further developed for regenerative therapy.

Effective decellularization that clears all cellular components, including cytoskeletal molecules and DNA, is essential to creating standardized and non-immunogenic cell type-specific CDMs for cell culture studies and therapeutic applications. However, the traditional NH_4_OH/TX-100/DNase cell extraction method that was originally developed for cultures of mouse embryonic fibroblasts^27^ resulted in incomplete removal of cytoskeletal filamentous actin and β-tubulin from the human gingival fibroblast cultures. Latrunculin B, an inhibitor of actin polymerization and destabilizer of actin filaments^90,91^, has been used previously for effective decellularization of mouse and chicken muscle tissue^25,92^, but it has not been tested with human fibroblast cultures. Our findings showed that latrunculin B followed by sodium deoxycholate and DNase treatment was efficient in removing cells, actin, β-tubulin, and DNA from the cultures. Further analysis of the CDMs generated from MMC and nMMC cultures using this decellularization method showed that the above differences noted in accumulation of the individual ECM molecules in the cultures containing cells were replicated also in the corresponding CDMs. Thus, MMC and the novel decellularization method can be used to generate distinct fibroblast-derived CDMs for further applications. However, we also noted considerable total protein loss (up to > 80%) due to decellularization in both MMC and nMMC cultures. This is not surprising considering that the cultures were composed of ECM and layers of cells that were growing in high density. Therefore, removing cells, and likely also cell-associated molecules, by decellularization is expected to cause dramatic reduction of total proteins in the cultures. Interestingly, protein loss was distinct for different ECM molecules. For instance, almost 80% of cellular fibronectin, but only about 20% of tenascin C, was lost due to decellularization in day 14 cultures. This difference could be explained by distinct distribution of cellular fibronectin and tenascin C in intracellular, cell surface, and ECM compartments and/or by different susceptibility of the ECM proteins to decellularization reagents. Interestingly, loss of collagen I was significantly decreased in older cultures compared to earlier time points, suggesting that collagen I fibrils become more stable over time. In addition, collagen I loss was significantly lower in MMC compared to nMMC cultures. This could be caused by stabilization of collagen fibrils by increase in activity of lysyl oxidase and transglutaminase 2 that have been shown to promote collagen crosslinking under MMC conditions^5^. In any case, based on previous studies^9,44,93,94^ and our findings, different decellularization protocols may display distinct efficacy to remove cytoskeletal proteins depending on cell type and culture conditions. In addition, fibroblast cultures generated with MMC may be partially protected from loss of fibronectin, collagen I and IV, and laminin 1.

The present proof-of-principle study provided first evidence of the utility of MMC technology in gingival fibroblast cultures. However, finding out tissue type-specific properties of the cultures requires analysis of parallel cell cultures isolated from multiple gingival tissue donors and their comparison to cultures with cells isolated from other tissues, such as skin. Future studies should also interrogate in more detail levels and function/activity of key proteins in cultures generated with MMC. These studies should also include large scale proteomes of human gingival fibroblast 3D cultures generated with MMC in comparison to the gingival tissue in vivo to characterize their similarities and differences in more detail. Based on our findings cell cultures and CDMs generated with MMC and the novel decellularization protocol could offer new tools to study in vivo-like cell-ECM interactions in vitro and provide a substitute for animal experiments. Furthermore, if MMC-derived CDMs recapitulate the above anti-fibrotic effect found in MMC cultures, they may have use as a less fibrotic wound healing model to study cell–matrix interactions. In addition, they could be developed into novel tissue-specific cell-derived biomaterials that can be used to promote scar-free wound healing.

## Supplementary Information

Supplementary Information.

## Data Availability

The datasets generated and/or analyzed during the current study are available from the corresponding author upon request.

## References

[1] Mouw JK, Ou G, Weaver VM (2014). Extracellular matrix assembly: A multiscale deconstruction. Nat. Rev. Mol. Cell Biol..

[2] Muhl L (2020). Single-cell analysis uncovers fibroblast heterogeneity and criteria for fibroblast and mural cell identification and discrimination. Nat. Commun..

[3] Ellis RJ (2001). Macromolecular crowding: Obvious but underappreciated. Trends Biochem. Sci..

[4] Ellis RJ, Minton AP (2003). Join the crowd. Nature.

[5] Chen C, Loe F, Blocki A, Peng Y, Raghunath M (2011). Applying macromolecular crowding to enhance extracellular matrix deposition and its remodeling in vitro for tissue engineering and cell-based therapies. Adv. Drug Deliv. Rev..

[6] Satyam A, Kumar P, Cigognini D, Pandit A, Zeugolis DI (2016). Low, but not too low, oxygen tension and macromolecular crowding accelerate extracellular matrix deposition in human dermal fibroblast culture. Acta Biomater..

[7] Tsiapalis D, Zeugolis DI (2021). It is time to crowd your cell culture media—Physicochemical considerations with biological consequences. Biomaterials.

[8] Wong AP (2019). Conversion of human and mouse fibroblasts into lung-like epithelial cells. Sci. Rep..

[9] Benny P, Badowski C, Lane EB, Raghunath M (2015). Making more matrix: Enhancing the deposition of dermal–epidermal junction components in vitro and accelerating organotypic skin culture development, using macromolecular crowding. Tissue Eng. Part A.

[10] Kaltschmidt B, Kaltschmidt C, Widera D (2012). Adult craniofacial stem cells: Sources and relation to the neural crest. Stem Cell Rev. Rep..

[11] Häkkinen L, Larjava H, Fournier BPJ (2014). Distinct phenotype and therapeutic potential of gingival fibroblast. Cytotheraphy.

[12] Longaker MT (1994). Adult skin wounds in the fetal environment heal with scar formation. Ann. Surg..

[13] Mak K (2009). Scarless healing of oral mucosa is characterized by faster resolution of inflammation and control of myofibroblast action compared to skin wounds in the red Duroc pig model. J. Dermatol. Sci..

[14] Wong JW (2009). Wound healing in oral mucosa results in reduced scar formation as compared with skin: Evidence from the red duroc pig model and humans. Wound Repair Regen..

[15] Glim JE, Van Egmond M, Niessen FB, Everts V, Beelen RHJ (2013). Detrimental dermal wound healing: What can we learn from the oral mucosa?. Wound Repair Regen..

[16] Mah W (2014). Human gingival fibroblasts display a non-fibrotic phenotype distinct from skin fibroblasts in three-dimensional cultures. PLoS ONE.

[17] Guo F, Carter DE, Mukhopadhyay A, Leask A (2011). Gingival fibroblasts display reduced adhesion and spreading on extracellular matrix: A possible basis for scarless tissue repair?. PLoS ONE.

[18] Mah W (2017). Elevated CD26 expression by skin fibroblasts distinguishes a profibrotic phenotype involved in scar formation compared to gingival fibroblasts. Am. J. Pathol..

[19] Fissell WH (2007). Ficoll is not a rigid sphere. Am. J. Physiol. Ren. Physiol..

[20] Fissell WH, Hofmann CL, Smith R, Chen MH (2010). Size and conformation of Ficoll as determined by size-exclusion chromatography followed by multiangle light scattering. Am. J. Physiol. Ren. Physiol..

[21] Hakkinen L, Heino J, Koivisto L, Larjava H (1994). Altered interaction of human granulation-tissue fibroblasts with fibronectin is regulated by α5β1 integrin. Biochim. Biophys. Acta.

[22] Cukierman E, Pankov R, Stevens DR, Yamada KM (2001). Taking cell-matrix adhesions to the third dimension. Science.

[23] Lareu RR (2007). Collagen matrix deposition is dramatically enhanced in vitro when crowded with charged macromolecules: The biological relevance of the excluded volume effect. FEBS Lett..

[24] Bi J (2016). Epithelial microvesicles promote an inflammatory phenotype in fibroblasts. J. Dent. Res..

[25] Gillies AR, Smith LR, Lieber RL, Varghese S (2011). Method for decellularizing skeletal muscle without detergents or proteolytic enzymes. Tissue Eng. Part C.

[26] Chaturvedi V (2015). Interactions between skeletal muscle myoblasts and their extracellular matrix revealed by a serum free culture system. PLoS ONE.

[27] Franco-Barraza J, Beacham DA, Amatangelo MD, Cukierman E (2017). Preparation of extracellular matrices produced by cultured and primary fibroblasts. Curr. Protoc. Cell Biol..

[28] Uhal BD (1998). Cell size, cell cycle, and α-smooth muscle actin expression by primary human lung fibroblasts. Am. J. Physiol. Lung Cell. Mol. Physiol..

[29] Neumann FR, Nurse P (2007). Nuclear size control in fission yeast. J. Cell Biol..

[30] Chen B, Co C, Ho C (2015). Cell shape dependent regulation of nuclear morphology. Biomaterials.

[31] Wu Y, Pegoraro AF, Weitz DA, Janmey P, Sun SX (2022). The correlation between cell and nucleus size is explained by an eukaryotic cell growth model. PLOS Comput. Biol..

[32] Gürses N, Thorup AK, Reibel J, Carter WG, Holmstrup P (1999). Expression of VLA-integrins and their related basement membrane ligands in gingiva from patients of various periodontitis categories. J. Clin. Periodontol..

[33] Bachmann M, Kukkurainen S, Hytönen VP, Wehrle-Haller B (2019). Cell adhesion by integrins. Physiol. Rev..

[34] Lu P, Takai K, Weaver VM, Werb Z (2011). Extracellular matrix degradation and remodeling in development and disease. Cold Spring Harb. Perspect. Biol..

[35] Melendez-Zajgla J, Del Pozo L, Ceballos G, Maldonado V (2008). Tissue Inhibitor of Metalloproteinases-4. The road less traveled. Mol. Cancer.

[36] Semenza GL (2016). The hypoxic tumor microenvironment: A driving force for breast cancer progression. Biochim. Biophys. Acta.

[37] Liakouli V (2011). Angiogenic cytokines and growth factors in systemic sclerosis. Autoimmun. Rev..

[38] Klingberg F, Hinz B, White ES (2013). The myofibroblast matrix: Implications for tissue repair and fibrosis. J. Pathol..

[39] Midwood KS, Orend G (2009). The role of tenascin-C in tissue injury and tumorigenesis. J. Cell Commun. Signal..

[40] Bradshaw AD (2009). The role of SPARC in extracellular matrix assembly. J. Cell Commun. Signal..

[41] Pakshir P (2020). The myofibroblast at a glance. J. Cell Sci..

[42] Vlodavsky I (2001). Preparation of extracellular matrices produced by cultured corneal endothelial and PF-HR9 endodermal cells. Curr. Protoc. Cell Biol..

[43] Kumar P (2015). Macromolecularly crowded in vitro microenvironments accelerate the production of extracellular matrix-rich supramolecular assemblies. Sci. Rep..

[44] Wong CW (2019). In vitro expansion of keratinocytes on human dermal fibroblast-derived matrix retains their stem-like characteristics. Sci. Rep..

[45] Gaspar D, Ryan CNM, Zeugolis DI (2019). Multifactorial bottom-up bioengineering approaches for the development of living tissue substitutes. FASEB J..

[46] Fan C (2019). Application of “macromolecular crowding” in vitro to investigate the naphthoquinones shikonin, naphthazarin and related analogues for the treatment of dermal scars. Chem. Biol. Interact..

[47] Satyam A, Tsokos MG, Tresback JS, Zeugolis DI, Tsokos GC (2020). Cell-derived extracellular matrix-rich biomimetic substrate supports podocyte proliferation, differentiation, and maintenance of native phenotype. Adv. Funct. Mater..

[48] Shendi D (2019). Hyaluronic acid as a macromolecular crowding agent for production of cell-derived matrices. Acta Biomater..

[49] Chester D (2022). Elucidating the combinatorial effect of substrate stiffness and surface viscoelasticity on cellular phenotype. J. Biomed. Mater. Res. Part A.

[50] Muncie, J. M. & Weaver, V. M. The physical and biochemical properties of the extracellular matrix regulate cell fate. in *Current Topics in Developmental Biology* vol. 130 1–37 (Elsevier Inc., 2018). 10.1016/bs.ctdb.2018.02.002 PMC6586474 29853174

[51] Geiger B, Bershadsky A, Pankov R, Yamada KM (2001). Transmembrane extracellular matrix cytoskeleton crosstalk. Nat. Rev. Mol. Cell Biol..

[52] Bornstein P, McPherson J, Sage H, Nossel HL, Vogel HJ (1982). Synthesis and secretion of structural macromolecules by endothelial cells in culture. Pathobiology of the Endothelial Cell.

[53] Bissell MJ, Hall HG, Parry G (1982). How does the extracellular matrix direct gene expression?. J. Theor. Biol..

[54] Fang F (2013). Early growth response 3 (Egr-3) is induced by transforming growth factor-β and regulates fibrogenic responses. Am. J. Pathol..

[55] Sethi A, Mao W, Wordinger RJ, Clark AF (2011). Transforming growth factor–B induces extracellular matrix protein cross-linking lysyl oxidase (LOX) genes in human trabecular meshwork cells. Investig. Ophthalmol. Vis. Sci..

[56] Cabral-Pacheco GA (2020). The roles of matrix metalloproteinases and their inhibitors in human diseases. Int. J. Mol. Sci..

[57] Yue J, Zhang K, Chen JF (2012). Role of integrins in regulating proteases to mediate extracellular matrix remodeling. Cancer Microenviron..

[58] Pulkkinen L (1995). Cloning of the β3 chain gene (LAMB3) of human laminin 5, a candidate gene in junctional Epidermolysis bullosa. Genomics.

[59] Zheng DQ (1997). Modulation of cell proliferation by the integrin cytoplasmic domain. Kidney Int..

[60] Kim SH, Turnbull J, Guimond S (2011). Extracellular matrix and cell signalling: The dynamic cooperation of integrin, proteoglycan and growth factor receptor. J. Endocrinol..

[61] El-Mohri H, Wu Y, Mohanty S, Ghosh G (2017). Impact of matrix stiffness on fibroblast function. Mater. Sci. Eng. C.

[62] Kollmannsberger P, Bidan CM, Dunlop JWC, Fratzl P, Vogel V (2018). Tensile forces drive a reversible fibroblast-to-myofibroblast transition during tissue growth in engineered clefts. Sci. Adv..

[63] Batt DB, Roberts TM (1998). Cell density modulates protein-tyrosine phosphorylation. J. Biol. Chem..

[64] Tarzemany R, Jiang G, Larjava H, Häkkinen L (2015). Expression and function of connexin 43 in Human gingival wound healing and fibroblasts. PLoS ONE.

[65] Schutz L, Mora PT (1968). The need for direct cell contact in “contact” inhibition of cell division in culture. J. Cell. Physiol..

[66] Rowe DW, Starman BJ, Fujimoto WY, Williams RH (1977). Abnormalities in proliferation and protein synthesis in skin fibroblast cultures from patients with diabetes mellitus. Diabetes.

[67] Lemons JMS (2010). Quiescent fibroblasts exhibit high metabolic activity. PLoS Biol..

[68] Agren MS, Steenfos HH, Dabelsteen S, Hansen JB, Dabelsteen E (1999). Proliferation and mitogenic response to PDGF-BB of fibroblasts isolated from chronic venous leg ulcers is ulcer-age dependent. J. Invest. Dermatol..

[69] Li X (2020). Establishment and biological characteristics of fibroblast cell lines obtained from wild corsac fox. Vitr. Cell. Dev. Biol. Anim..

[70] Umeno Y, Okuda A, Kimura G (1989). Proliferative behaviour of fibroblasts in plasma-rich culture medium. J. Cell Sci..

[71] Iyer VR (1999). The transcriptional program in the response of human fibroblasts to serum. Science.

[72] Thomas CH, Collier JH, Sfeir CS, Healy KE (2002). Engineering gene expression and protein synthesis by modulation of nuclear shape. Proc. Natl. Acad. Sci. USA.

[73] Kutys ML, Doyle AD, Yamada KM (2013). Regulation of cell adhesion and migration by cell-derived matrices. Exp. Cell Res..

[74] Chang HY (2004). Gene expression signature of fibroblast serum response predicts human cancer progression: Similarities between tumors and wounds. PLoS Biol..

[75] Doolin MT, Smith IM, Stroka KM (2021). Fibroblast to myofibroblast transition is enhanced by increased cell density. Mol. Biol. Cell.

[76] Satyam A (2014). Macromolecular crowding meets tissue engineering by self-assembly: A paradigm shift in regenerative medicine. Adv. Mater..

[77] Li J (2022). Spatially resolved proteomic map shows that extracellular matrix regulates epidermal growth. Nat. Commun..

[78] Buskermolen JK (2016). Development of a full-thickness human gingiva equivalent constructed from immortalized keratinocytes and fibroblasts. Tissue Eng. Part C Methods.

[79] Roffel S (2019). Evaluation of a novel oral mucosa in vitro implantation model for analysis of molecular interactions with dental abutment surfaces. Clin. Implant Dent. Relat. Res..

[80] Rousselle P, Scoazec JY (2020). Laminin 332 in cancer: When the extracellular matrix turns signals from cell anchorage to cell movement. Semin. Cancer Biol..

[81] Kriegebaum U (2012). Tissue engineering of human oral mucosa on different scaffolds: In vitro experiments as a basis for clinical applications. Oral Surg. Oral Med. Oral Pathol. Oral Radiol..

[82] Nissen NI, Karsdal M, Willumsen N (2019). Collagens and Cancer associated fibroblasts in the reactive stroma and its relation to Cancer biology. J. Exp. Clin. Cancer Res..

[83] Kulasekara KK (2009). Cancer progression is associated with increased expression of basement membrane proteins in three-dimensional in vitro models of human oral cancer. Arch. Oral Biol..

[84] Fullár A (2015). Remodeling of extracellular matrix by normal and tumor-associated fibroblasts promotes cervical cancer progression. BMC Cancer.

[85] Chen X, Song E (2019). Turning foes to friends: Targeting cancer-associated fibroblasts. Nat. Rev. Drug Discov..

[86] Pastar I (2014). Epithelialization in wound healing: A comprehensive review. Adv. Wound Care.

[87] Tsutsui K (2021). Mapping the molecular and structural specialization of the skin basement membrane for inter-tissue interactions. Nat. Commun..

[88] Jayadev R, Sherwood DR (2017). Basement membranes. Curr. Biol..

[89] Yu G (2016). Gingival fibroblasts as autologous feeders for induced pluripotent stem cells. J. Dent. Res..

[90] Gibbon BC, Kovar DR, Staiger CJ (1999). Latrunculin B has different effects on pollen germination and tube growth. Plant Cell.

[91] Pollard TD, Earnshaw WC, Johnson GT, Pollard TD, Earnshaw WC, Johnson GT, Lippincott-Schwartz J (2017). Actin and actin-binding proteins. Cell Biology.

[92] Reyna WE, Pichika R, Ludvig D, Perreault EJ (2020). Efficiency of skeletal muscle decellularization methods and their effects on the extracellular matrix. J. Biomech..

[93] Xing Q (2015). Decellularization of fibroblast cell sheets for natural extracellular matrix scaffold preparation. Tissue Eng. Part C Methods.

[94] Parmaksiz M, Elçin AE, Elçin YM (2020). Decellularized cell culture ECMs act as cell differentiation inducers. Stem Cell Rev. Rep..

