# Supplementary material for: Macromolecular crowding regulates matrix composition and gene expression in human gingival fibroblast cultures
Source: Sci Rep. 2023 Feb 4;13:2047. doi: 10.1038/s41598-023-29252-1 (PMC9899282; doi:10.1038/s41598-023-29252-1)

**SUPPLEMENTARY INFORMATION**

**Supplementary Materials and Methods**

**Supplementary Table S1. List of primers used for RT-qPCR.**

| **GeneBank** | **Gene** | **Primer sequence** | **Orientation** | **Location** | **Amplicon (bp)** |
| --- | --- | --- | --- | --- | --- |
| **Fibrillar Protein Extracellular Matrix Molecules** | | | | | |
| BC036531 | *COL1A1* - Collagen Type 1 (alpha 1) | AACCAAGGCTGCAACCTGGA | Forward | 3951-3970 | 80 |
|  |  | GGCTGAGTAGGGTACACGCAGG | Reverse | 4030-4009 |  |
| NM_000090 | *COL3A1* - Collagen Type 3 (alpha 1) | CTCCTGGGATTAATGGTAGT | Forward | 1271-1290 | 70 |
|  |  | CCAGGAGCTCCAGGAAT | Reverse | 1340-1324 |  |
| NM_000093 | *COL5A1* - Collagen Type 5 (alpha 1) | GAGGACTGGGTTGTGCCTA | Forward | 7202-7220 | 120 |
|  |  | TAGCACCACAAACATTCAGGAA | Reverse | 7322-7301 |  |
| **Basement Membrane Associated Extracellular Matrix Molecules** | | | | | |
| NM_005559 | *LAMA1* - Laminin (alpha1) | TTGACCTTGGCAAAGGCA | Forward | 8424~8441 | 86 |
|  |  | TAACATAGTCTGTCTTGACCGTG | Reverse | 8510~8488 |  |
| NM_001017402 | *LAMB3* - Laminin 5 (beta subunit 3) | CTCCGAGCTTCATCTACCTG | Forward | 511~530 | 92 |
|  |  | GGAGTCACACTTGCAGCA | Reverse | 603~586 |  |
| NM_001845 | *COL4A1* - Collagen 4 (alpha 1) | AATTCCAGGGCAGCCAG | Forward | 1460~1476 | 85 |
|  |  | ATCCGTCTATATCACAGATGAGG | Reverse | 1545~1523 |  |
| NM_000094 | *COL7A1* - Collagen 7 (alpha 1) | TGGCAGCAGGGGAGAG | Forward | 7592-7607 | 86 |
|  |  | CCCAGGATCACAGCTGAGT | Reverse | 7678-7660 |  |
| **Glycoprotein Extracellular Matrix Molecules** | | | | | |
| NM_212482 | *EDA-FN1* - EDA Fibronectin | CACAGTCAGTGTGGTTGCCT | Forward | 5633-5652 | 68 |
|  |  | CTGTGGACTGGGTTCCAATCA | Reverse | 5700-5680 |  |
| NM_000501 | *ELN* - Elastin | GGGTCCCTGGGGCCATTC | Forward | 174~191 | 62 |
|  |  | CAGGCCCCAGCGCTGGAT | Reverse | 236~219 |  |
| NM_000138 | *FBN1* - Fibrillin 1 | AGGAAACGGAGAAGCACAA | Forward | 8577~8595 | 103 |
|  |  | CTGTCTTCTCAACATCCCAA | Reverse | 8679~8660 |  |
| NM_003118 | *SPARC* - Osteonectin | ATCCCTGCCAGAACCACCACT | Forward | 314~334 | 97 |
|  |  | TGGGCAGCTGGTGGGGT | Reverse | 411~395 |  |
| NM_006475 | *POSTN* - Periostin | ACACGAGAAGAACGAATCAT | Forward | 2363~2382 | 97 |
|  |  | GTAACAATTTCTTCAGAGTTTCTTC | Reverse | 2460~2435 |  |
| NM_003246 | *THBS1*- Thrombospondin 1 | CATCTTGTTCTGTGACATGTGG | Forward | 1513~1534 | 92 |
|  |  | TTCACAGGGTTTCCCGTTC | Reverse | 1604~1586 |  |
| NM_003247 | *THBS2*- Thrombospondin 2 | TTGTGTTCAACCCAGACCA | Forward | 2989~3007 | 107 |
|  |  | CAGGACACACATCATCAATATC | Reverse | 3096~3075 |  |
| NM_002160 | *TNC* - Tenascin C | CAACCTGATGGGGAGATATGGGGA | Forward | 6769-6792 | 75 |
|  |  | GAGTGTTCGTGGCCCTTCCAG | Reverse | 6846-6826 |  |
| NM_019105 | *TNX* - Tenascin X | GCATGGATGGACAGACAG | Forward | 12420~12437 | 71 |
|  |  | CCAAAACCATGGGCATAGTC | Reverse | 12477~12458 |  |
| **Small Leucine-rich Proteolgycan Glycoprotein Extracellular Matrix Molecules** | | | | | |
| NM_001711 | *BGN* - Biglycan | CTCAAGCTCCTCCAGGTGGTC | Forward | 1067-1087 | 93 |
|  |  | CCGAAGCCCATGGGACAGAAGTC | Reverse | 1151-1127 |  |
| BT019800 | *DCN* - Decorin | CTGACACAACTCTGCTAGAC | Forward | 242-261 | 97 |
|  |  | GACAAGAATCAATGCGTGAAG | Reverse | 339-319 |  |
| BT006707 | *LUM* - Lumican | TAGACAACAATAAGATCAGCAACA | Forward | 635-658 | 85 |
|  |  | TTCGTTGTGAGATAAACGCAG | Reverse | 720-700 |  |
| NM_002023 | *FMOD* - Fibromodulin | CACAATGAGATCCAGGAAG | Forward | 761-779 | 85 |
|  |  | TCCGAAGGTGGTTATAACTC | Reverse | 845-826 |  |
| **Regulation of ECM Synthesis Associated Enzymes** | | | | | |
| NM_005328 | *HAS2* - Hyaluronan Synthase 2 | CTAAACCAGCAGACCCGTTG | Forward | 1580~1599 | 145 |
|  |  | TGGATTACTGTGGCAATGAGAA | Reverse | 1725~1704 |  |
| NM_005329 | *HAS3* - Hyaluronan Synthase 3 | CGATTCGGTGGACTACATCCA | Forward | 771~791 | 90 |
|  |  | ATCCTCCTCCAGGACTCGA | Reverse | 861~843 |  |
| NM_018357 | *LARP6* - La Ribonucleoprotein 6 | ACAAGCTCCACTGAAAACCAC | Forward | 1564-1584 | 154 |
|  |  | TCAAACCATGGCGTACCGA | Reverse | 1718-1700 |  |
| **Matrix Metalloproteinases and Tissue Inhibitors of Metalloproteinases** | | | | | |
| NM_002421 | *MMP1* - Matrix Metalloproteinase 1 | GCTAACAAATACTGGAGGTATGATG | Forward | 1250-1275 | 100 |
|  |  | GTCATGTGCTATCATTTTGGGA | Reverse | 1304-1325 |  |
| NM_001166308 | *MMP3* - Matrix Metalloproteinase 3 | ATGATGAACAATGGACAAAGGA | Forward | 661-682 | 91 |
|  |  | GAGTGAAAGAGACCCAGGGA | Reverse | 751-732 |  |
| NM_002423 | *MMP7* - Matrix Metalloproteinase 7 | CCAGATGTTGCAGAATACTCACTA | Forward | 315~338 | 85 |
|  |  | GAGTATATGATACGATCCTGTAGGTG | Reverse | 399~374 |  |
| NM_002425 | *MMP10* - Matrix Metalloproteinase 10 | TTATACACCAGATTTGCCAAGA | Forward | 394-415 | 56 |
|  |  | TTCAGAGCTTTCTCAATGG | Reverse | 450-432 |  |
| NM_005940 | *MMP11* - Matrix Metalloproteinase 11 | CTATCCTCCAAAGCCATTGTAA | Forward | 2258~2179 | 106 |
|  |  | CAACTGTGTTTAATGACAATCCTC | Reverse | 2254~2231 |  |
| NM_003254 | *TIMP1* - Tissue Inhibitor of Metalloproteinase 1 | CTGTGTCCCACCCCACC | Forward | 267~283 | 64 |
|  |  | GAACTTGGCCCTGATGACGA | Reverse | 330~311 |  |
| NM_003255 | *TIMP2* - Tissue Inhibitor of Metalloproteinase 1 | ACATTTATGGCAACCCTATCAA | Forward | 481~502 | 70 |
|  |  | TCAGGCCCTTTGAACATCTTTA | Reverse | 550~529 |  |
| NM_000362 | *TIMP3* - Tissue Inhibitor of Metalloproteinase 1 | AGGACGCCTTCTGCAAC | Forward | 1281~1297 | 68 |
|  |  | CTCCTTTACCAGCTTCTTCC | Reverse | 1348~1329 |  |
| NM_003256 | *TIMP4* - Tissue Inhibitor of Metalloproteinase 1 | ACCTGTCCTTGGTGCAGA | Forward | 927~944 | 80 |
|  |  | TGTAGCAGGTGGTGATTTGG | Reverse | 1004~985 |  |
| **ECM Crosslinking Enzymes** | | | | | |
| NM_002317 | LOX - Lysyl Oxidase | TCCTGGGAATGGCACAGTTG | Forward | 1127-1146 | 100 |
|  |  | CCTTCAGCCACTCTCCTCTGG | Reverse | 1227-1207 |  |
| NM_005576 | LOXL1 - Lysyl Oxidase Like 1 | TGGGAACTACATCCTCAAGGTG | Forward | 1906-1927 | 97 |
|  |  | CTGTGTAGTGAATGTTGCATCTCAC | Reverse | 2003-1979 |  |
| NM_002318 | LOXL2 - Lysyl Oxidase Like 2 | AAGTCCTGGACTGCCAGCTC | Forward | 566-585 | 86 |
|  |  | GGTCGCCTCGTTGCCAGTA | Reverse | 652-634 |  |
| NM_032603 | LOXL3 - Lysyl Oxidase Like 3 | TCAGTGGATTGACATCACGGA | Forward | 2107-2127 | 145 |
|  |  | GCACCCAGATTCTATGTCCATCA | Reverse | 2252-2230 |  |
| NM_032211 | LOXL4 - Lysyl Oxidase Like 4 | ACAACTGCCACACAGGGAA | Forward | 2281-2299 | 94 |
|  |  | GTGACAGCTTCAGATGAGGTTG | Reverse | 2375-2354 |  |
| NM_000302 | PLOD1 - Procollagen-Lysine,2-Oxoglutarate 5-Dioxygenase 1 | GCTGCTGAAGAAAGCTCTGG | Forward | 300-319 | 78 |
|  |  | CAGCACGTCATAGCTGTCTG | Reverse | 378-359 |  |
| NM_000935 | PLOD2 - Procollagen-Lysine,2-Oxoglutarate 5-Dioxygenase 2 | TGTTCAAGGGAATAGAGTAGGAG | Forward | 1536-1558 | 141 |
|  |  | GCAAAGAGCCATATCAGGAT | Reverse | 1677-1658 |  |
| NM_001084 | PLOD3 - Procollagen-Lysine,2-Oxoglutarate 5-Dioxygenase 3 | TCCGAGGACTACGTGGAGC | Forward | 1628-1646 | 67 |
|  |  | TGGGAGATGTATGGTACATTCCA | Reverse | 1695-1673 |  |
| **Integrins (Cell Adhesion Molecules)** | | | | | |
| NM_181501 | *ITGA1* - Integrin Alpha 1 | GATGTGGACATTTGCATTACAC | Forward | 848-869 | 103 |
|  |  | CAGTTGAGTGCTGCATTCTT | Reverse | 951-932 |  |
| NM_002203 | *ITGA2* - Integrin Alpha 2 | TGGTTCAATGTTGAAAGCTGTG | Forward | 981-1002 | 80 |
|  |  | AAGTACCCAAGAACTGCTATGC | Reverse | 1061-1040 |  |
| NM_002204 | *ITGA3* - Integrin Alpha 3 | GCAAGGAGTGGGACTTATCTGA | Forward | 1016-1037 | 96 |
|  |  | AGGATGAAGCTGCCTACCTG | Reverse | 1112-1093 |  |
| NM_000885 | *ITGA4* - Integrin Alpha 4 | AGCTGGGTAGCCCTAATGGA | Forward | 583-602 | 126 |
|  |  | CTATGCCCACAAGTCACGATGG | Reverse | 709-688 |  |
| NM_002205 | *ITGA5* - Integrin Alpha 5 | CCAAGGAGGCTTCAGTGC | Forward | 683-700 | 77 |
|  |  | CCTTGCCAGAAATAGCTTCCTG | Reverse | 760-739 |  |
| NM_000210 | *ITGA6* - Integrin Alpha 6 | CCGTATGATGACTTGGGAAAGG | Forward | 1401-1422 | 133 |
|  |  | CGATCAAGGTCCATGTTTCCAG | Reverse | 1534-1513 |  |
| NM_002206 | *ITGA7* - Integrin Alpha 7 | AGGAGCTGCATCCAGTCTCT | Forward | 2591-2610 | 98 |
|  |  | ACCAGAGAAGAAGAGTTGCTGG | Reverse | 2688-2667 |  |
| NM_003638 | *ITGA8* - Integrin Alpha 8 | ACAGTTTGGACGAATCCACCT | Forward | 1968-1988 | 106 |
|  |  | ACAGTCCACCAGAATGTGAGC | Reverse | 2074-2054 |  |
| NM_002207 | *ITGA9* - Integrin Alpha 9 | TGGGGATGGTGCCTACAATG | Forward | 1299-1318 | 108 |
|  |  | GTCATCCTCCTTGGGTGCA | Reverse | 1407-1389 |  |
| NM_003637 | *ITGA10* - Integrin Alpha 10 | AGCAGTGACAGCCTGGAGA | Forward | 2776-2794 | 109 |
|  |  | TGCAGGGTAGACTCACTAGAGAAC | Reverse | 2885-2862 |  |
| NM_001004439 | *ITGA11* - Integrin Alpha 11 | GAAGGCACCAACAAGAACGA | Forward | 1144~1163 | 60 |
|  |  | AGGAAAAGCCCGTCTGTGA | Reverse | 1204~1186 |  |
| NM_002210 | *ITGAV* - Integrin Alpha V | GAGGAAAGAGTGCAATCTTGTA | Forward | 2930-2951 | 103 |
|  |  | GAAGCAGACGACTTCAGAGA | Reverse | 3032-3013 |  |
| NM_002211 | *ITGB1* - Integrin Beta 1 | ATGCCAAATCATGTGGAGAATG | Forward | 313~334 | 116 |
|  |  | GGCTTCTAAATCATCACATCGTG | Reverse | 428~406 |  |
| NM_000212 | *ITGB3* - Integrin Beta 3 | CTGCACCTTTAAGAAAGAATG | Forward | 1919-1939 | 122 |
|  |  | TGTCCTTAAGCTCTTTCACTG | Reverse | 2040-2020 |  |
| NM_000213 | *ITGB4* - Integrin Beta 4 | CGTGTGAGGAATGCAACTTCAAGG | Forward | 2127~2150 | 64 |
|  |  | ACCACCTCCTCGGCTCT | Reverse | 2190~2174 |  |
| NM_002213 | *ITGB5* - Integrin Beta 5 | TACAGCCCTGATACCTGGAAC | Forward | 1341-1361 | 107 |
|  |  | ACTCCACTTTAGACCGGATACTAT | Reverse | 1447-1424 |  |
| NM_000889 | *ITGB7* - Integrin Beta 7 | CAGGAGCTGAGTAAACTGATTCC | Forward | 1368-1390 | 120 |
|  |  | GAGGGAGTGAAGAGTGTTCAAG | Reverse | 1380-1359 |  |
| NM_002214 | *ITGB8* - Integrin Beta 8 | CTGCAAGGAAAACTGGAATTGTATG | Forward | 2604-2628 | 144 |
|  |  | CTTGGGCTGGAGAAACATTCTG | Reverse | 2747-2726 |  |
| **TGF-Beta Signaling (Growth Factor Signaling)** | | | | | |
| NM_000660 | *TGFB1* - Transforming Growth Factor Beta 1 | CAACGAAATCTATGACAAGTTCAAGCAG | Forward | 1218-1245 | 76 |
|  |  | CTTCTCGGAGCTCTGATGTG | Reverse | 1294-1275 |  |
| NM_003238 | *TGFB2* - Transforming Growth Factor Beta 2 | TGGTGAAAGCAGAGTTCAGAG | Forward | 1883~1903 | 140 |
|  |  | CACAACTTTGCTGTCGATGTAG | Reverse | 2022~2001 |  |
| NM_003239 | *TGFB3* - Transforming Growth Factor Beta 3 | ACACCAATTACTGCTTCCGCAA | Forward | 1161-1182 | 81 |
|  |  | GCCTAGATCCTGTCGGAAGTC | Reverse | 1242-1220 |  |
| NM_000627 | *LTBP1* - Latent TGF-beta Binding Protein 1 | AGGGTCCTATGATTGTACTTGTC | Forward | 2706~2728 | 94 |
|  |  | ACAGAGCCCTGGATGTTC | Reverse | 2799~2782 |  |
| NM_000428 | *LTBP2* - Latent TGF-beta Binding Protein 2 | AGGCGAGTGCAAGAACAC | Forward | 3831~3848 | 84 |
|  |  | TCACACACGGTGCCATTG | Reverse | 3911~3894 |  |
| NM_021070 | *LTBP3* - Latent TGF-beta Binding Protein 3 | CCACCACAAGAAGGAGTGCTAC | Forward | 3008~3029 | 84 |
|  |  | TCCTGCTGGGTCACGTTG | Reverse | 3091~3074 |  |
| NM_001042545 | *LTBP4* - Latent TGF-beta Binding Protein 4 | AGCTGCCCTGTGTGAAAATG | Forward | 3125~3144 | 82 |
|  |  | TCCAGTCATGGGGTCAAACT | Reverse | 3206~3187 |  |
| NM_001136179 | *EGR2* - Early Growth Response Protein 2 | AGCTTTGCTCCCGTCTCTG | Forward | 469~487 | 88 |
|  |  | AGCTGGCACCAGGGTACT | Reverse | 556~539 |  |
| NM_004430 | *EGR3* - Early Growth Response Protein 3 | CGTTGGACAGCAATCTCTTC | Forward | 903~922 | 75 |
|  |  | AATGGAGCCCATGTCGTTG | Reverse | 976~958 |  |
| NM_001142483 | *P311* - Neuronal Regeneration Related Protein | CCTGGACTGAAGAGAGG | Forward | 321~447 | 78 |
|  |  | CAGACAAAGAGTTCTGGGTA | Reverse | 508~489 |  |
| **Gap Junction Protein (Cell to Cell Communication)** | | | | | |
| NM_000165 | *GJA1* - Connexin 43 (Cx43) | AGCAGTCTGCCTTTCGTTGTA | Forward | 393-412 | 73 |
|  |  | GATTGGGAAAGACTTGTCATAGCAG | Reverse | 466-442 |  |
| **Profibrotic Fibroblast Lineage Markers** | | | | | |
| NM_001426 | *EN1* - Engrailed 1 | AGGGAAAAGAGCGAGAGAGAC | Forward | 2303-2323 | 156 |
|  |  | AAGGAGTTCGCAGTTTCGTC | Reverse | 2459-2440 |  |
| NM_001935 | *DPP4* - Dipeptidyl Peptidase 4 | AGTCGCAAAACTTACACTCTAACTG | Forward | 676~700 | 146 |
|  |  | GAGCTGTTTCCATATTCAGCATTG | Reverse | 821~798 |  |
| **Myofibroblast-Associated Molecules (Cell Contractility)** | | | | | |
| NM_001613 | *ACTA2* - Alpha Smooth Muscle Actin | AGCGTGGCTATTCCTTCGT | Forward | 637-655 | 97 |
|  |  | CTCATTTTCAAAGTCCAGAGCTACA | Reverse | 733-707 |  |
| NM_001318139 | *MKL1* - Myocardin Related Transcription Factor A | GCTGAAGAGAGCCAGACTAGC | Forward | 530-550 | 121 |
|  |  | CAATGATGGCTTCCTTCAGGC | Reverse | 651-631 |  |
| **Regulators of Angiogenesis** | | | | | |
| NM_001171630 | *VEGFA* - Vascular Endothelial Growth Factor A | AGTGTGTGCCCACTGAGGA | Forward | 1316-1334 | 97 |
|  |  | GTGCTGTAGGAAGCTCATCTC | Reverse | 1413-1393 |  |
| NM_199168 | *SDF1* - Stromal Cell Derived Factor 1 | TACAGATGCCCATGCCGA | Forward | 174-191 | 93 |
|  |  | CTGAAGGGCACAGTTTGGAG | Reverse | 266-247 |  |
| **Reference Genes** | | | | | |
| NM_002046 | *GAPDH* - Glyceraldehyde-3-Phosphate Dehydrogenase | CTTTGTCAAGCTCATTTCCTGGTA | Forward | 1020-1043 | 70 |
|  |  | GGCCATGAGGTCCACCA | Reverse | 1089-1073 |  |
| BC009255 | *ALG9* - Alpha-1,2-Mannosyltransferase | GAATGACCAGAATCTAGAAGAGCCA | Forward | 1668-1692 | 82 |
|  |  | TCTCATGGTGTCCAAATCCACTAAA | Reverse | 1749-1725 |  |
| NM_021009 | *UBC* - Ubiquitin C | GTGGCACAGCTAGTTCCGT | Forward | 371-389 | 96 |
|  |  | CTTCACGAAGATCTGCATTGTCA | Reverse | 444-467 |  |

**Supplementary Table S2. List of antibodies used.**

| **Antibody** | **Manufacturer** | **Catalog Number** | **Source** | **Dilution** |
| --- | --- | --- | --- | --- |
| Collagen I | abcam | ab34710 | Rabbit polyclonal | 1/200 IF |
| Collagen IV | Monosan | PS057 | Rabbit polyclonal | 1/200 IF |
| Alpha Smooth Muscle Actin | Sigma | A2547 | Mouse monoclonal (clone 1A4) | 1/200 IF |
|  | Santa Cruz | sc-32251 | Mouse (clone 1A4) | 1/200 WB |
| Actin | abcam | ab176757 | Phalloidin | 1/1000 IF |
| Beta Tubulin | Millipore | MAB3408 | Mouse monoclonal | 1/500 IF |
| Tenascin C | Sigma | T-2551 | Mouse monoclonal (clone BC-24) | 1/200 IF |
| EDA Fibronectin | abcam | ab6328 | Mouse monoclonal | 1/200 IF |
| Laminin 1 | Sigma | L9393 | Rabbit | 1/200 IF |
| LTBP1  (Latent TGF-β Binding Protein 1) | R&D Systems | MAB388 | Mouse monoclonal | 1/200 IF |
| SPARC (Osteonectin) | Santa Cruz Biotechnology | sc-25574 | Rabbit polyclonal | 1/100 IF |
| GAPDH | Santa Cruz Biotechnology | sc-25778 | Rabbit (clone FL-335) | 1/1000 WB |

IF: Immunofluorescence staining

WB: Western blotting

**Supplementary Results**

**Supplementary Figure S1. Thresholding of immunostaining for image analysis of αSMA stress fibers in MMC and nMMC cultures.** Panel shows representative standardized αSMA immunostaining images of 3- and 14-day MMC and nMMC cultures before (original image) and after thresholding (thresholded image) to differentiate αSMA stress fibers out of total αSMA immunostaining in the images. Magnification bar = 40 μm.


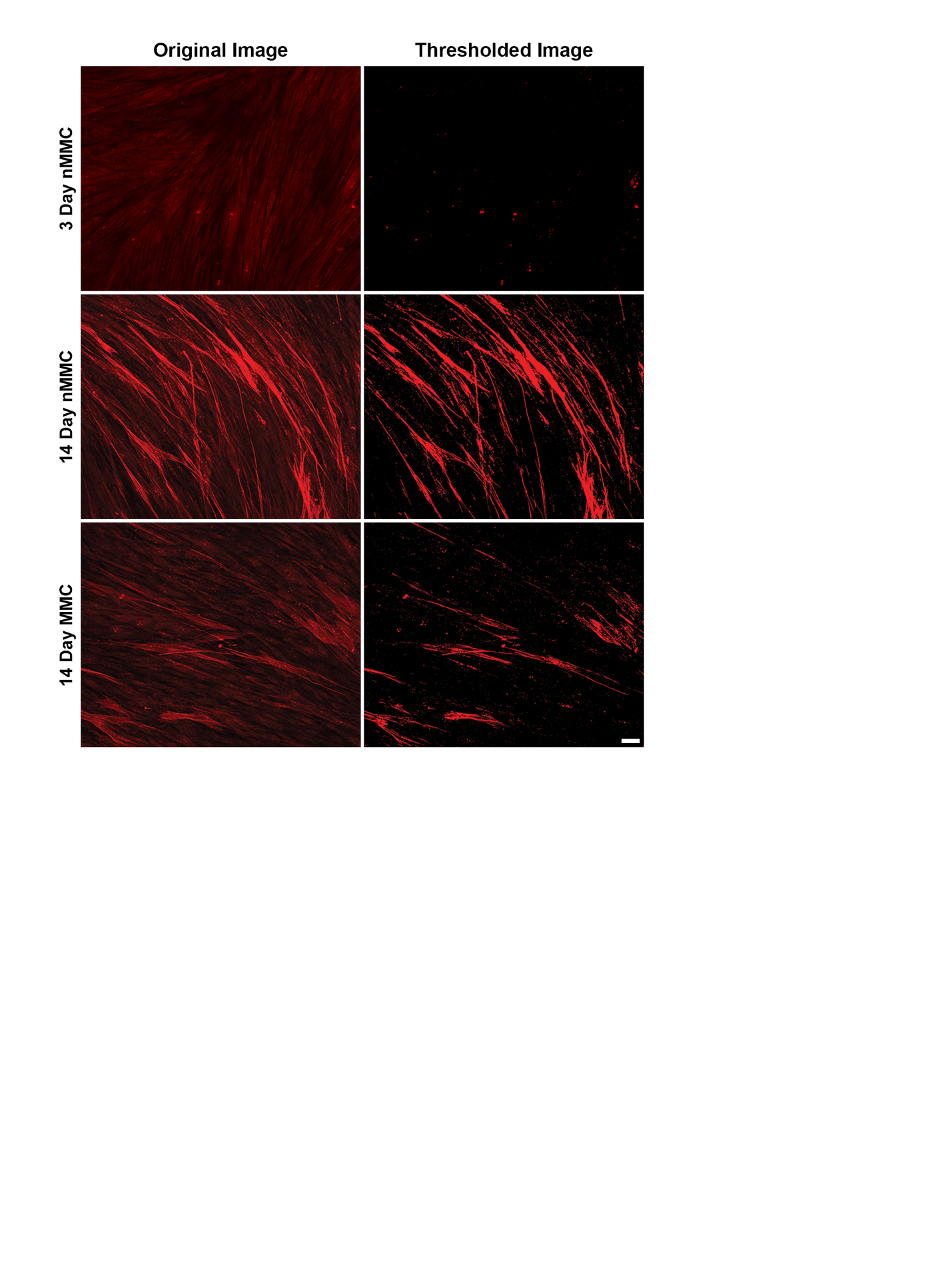


**Supplementary Figure S2. Analysis of fibroblast gene expression changes in MMC and nMMC cultures over time.** Table highlights statistically significant expression changes of a set of 32 genes over time analyzed by RT-qPCR. Cell color indicates significant downregulation (green) or upregulation (red) of mRNA expression compared to the earlier time point. Empty cell indicates no significant mRNA expression difference. For both culture conditions, expression of a set of genes changed significantly during the first 7 days of culture. However, for all genes tested, mRNA expression stabilized after day 7 (no significant gene expression changes were noted after that time point). For the experiments, GFBLs were cultured in MMC or nMMC condition for the indicated time before total RNA isolation. Day 0 indicates culture of cells for 24 hours in standard culture medium before change into fresh MMC or nMMC medium. Statistical testing was performed by one-way ANOVA (*p<0.05; **p<0.01; ***p<0.001), n=3 repeated experiments.


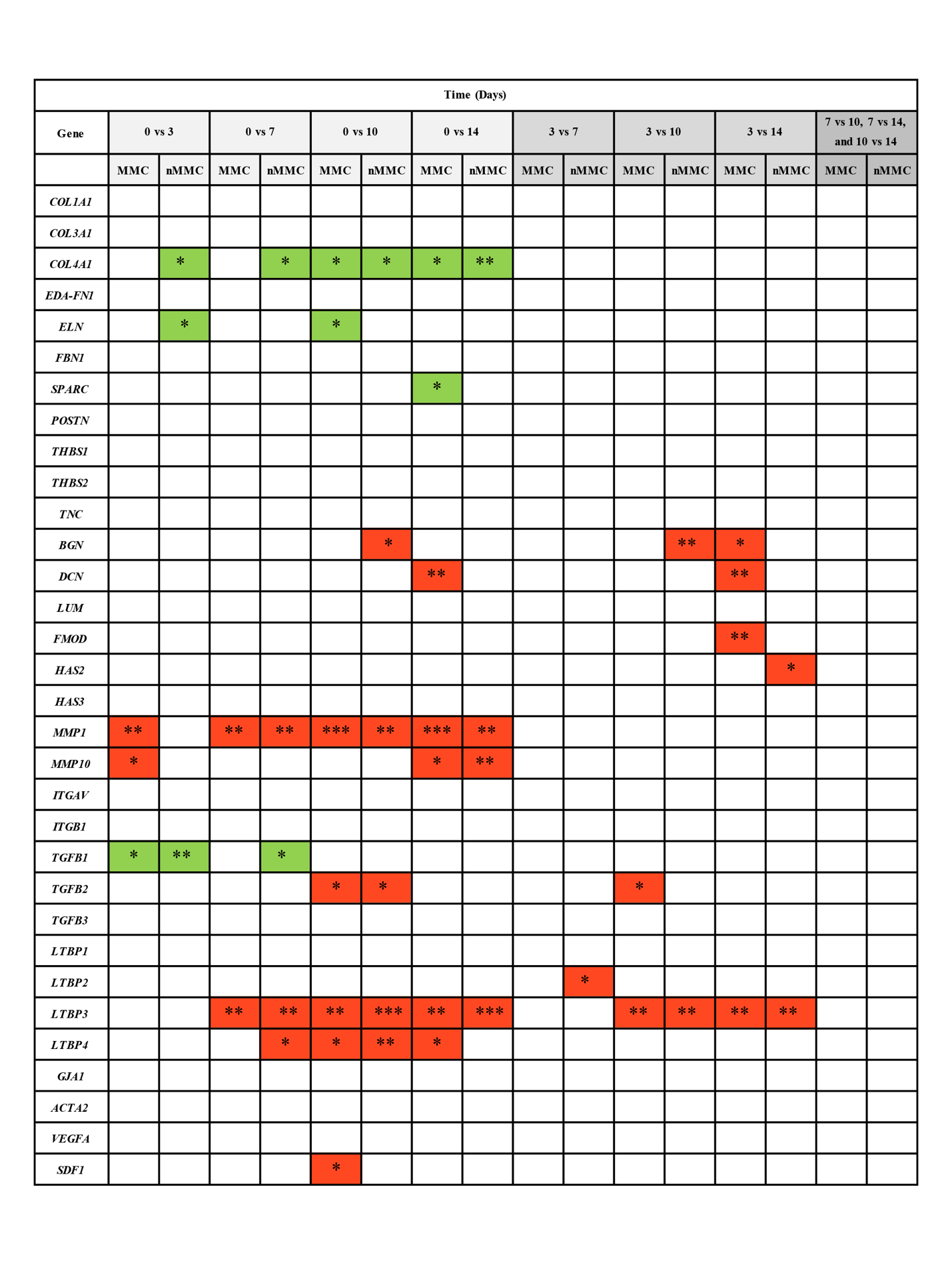


**Supplementary Figure S3. Quantification and characterization of collagen I organization and abundance in MMC and nMMC CDMs over time.**  **A)** Representative standardized images of collagen I immunostaining of CDMs from each time point and treatment from two repeated experiments. Inserts show higher magnification of select areas. Images in inserts are individually optimized for brightness/contrast. Magnification bars = 40 μm. **B)** Statistical comparison of change in total collagen I abundance in CDMs over time. **C)** Pairwise comparison of total collagen I abundance in CDMs at each time point. **D)** Statistical comparison of change in relative (normalized for cell numbers determined by nuclear DAPI counts in parallel samples) collagen I abundance in CDMs over time. **E)** Pairwise comparison of relative collagen I abundance in CDMs at each time point. Collagen I showed abundant fibrillar network already at day 3 CDMs from MMC cultures. In contrast, a sparce fibrillar network was evident only at day 10 and 14 CDMs from nMMC cultures (**A**). Similar to findings from cultures containing cells (Figure 4), quantitation of total collagen I showed significantly increased accumulation over time in both MMC and nMMC CDMs up to 14 days (**B and C**). However, when collagen I levels were normalized for cell numbers, they did not show marked changes in MMC and nMMC CDMs over time (**D and E**). Similar to findings from cultures containing cells (Figure 4), pairwise comparison of collagen I at each time point showed significantly higher levels of total (**C**) and relative (**E**) collagen I in MMC compared to nMMC CDMs at all time points. Results show mean +/- SEM **(C and E)** and statistical comparison over time (B and D) from image analysis performed with 6-12 images obtained from each time point and treatment from two repeated experiments. Statistical testing was performed by one-way ANOVA **(B and D)** and by independent samples t-test (**C and E**), *p<0.05; **p<0.01; ***p<0.001.


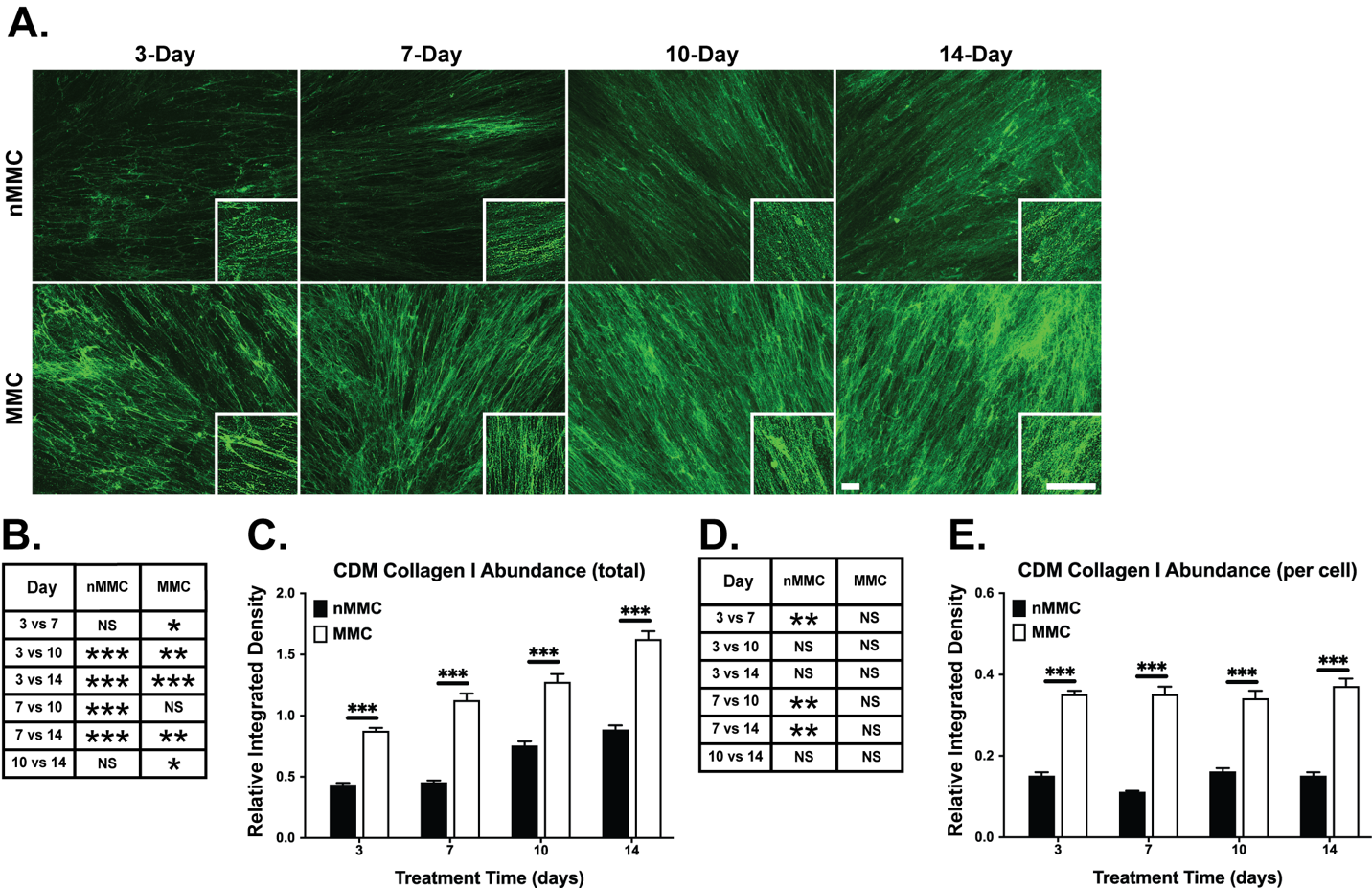


**Supplementary Figure S4. Characterization of collagen IV and cellular fibronectin organization and abundance in MMC and nMMC CDMs over time. A)** Representative standardized images of collagen IV immunostaining from each time point and treatment from two repeated experiments. Inserts show higher magnification of select areas. Images in inserts are individually optimized for brightness/contrast. **B)** Statistical comparison of change in total collagen IV abundance in CDMs over time. **C)** Pairwise comparison of total collagen IV abundance in CDMs at each time point. **D)** Statistical comparison of change in relative (normalized for cell numbers determined by nuclear DAPI counts in parallel samples) collagen IV abundance in CDMs over time. **E)** Pairwise comparison of relative collagen IV abundance in CDMs at each time point. Findings of collagen IV in CDMs also reflected those from cultures containing cells (Figure 5). Collagen IV showed abundant fibrillar network in CDMs from MMC cultures at day 7-14, while this was less apparent in nMMC CDMs even after 14 days (**A**). Quantification of total (**B and C**) and relative (**D and E**) collagen IV in CDMs showed significantly increased levels in CDMs from MMC compared to nMMC cultures at all time points. **F)** Representative standardized images of cellular fibronectin (EDA-fibronectin) immunostaining from each time point and treatment from two repeated experiments. Inserts show higher magnification of select areas. Images in inserts are individually optimized for brightness/contrast. **G)** Statistical comparison of change in total cellular fibronectin abundance in CDMs over time. **H)** Pairwise comparison of total cellular fibronectin abundance in CDMs at each time point. **I)** Statistical comparison of change in relative (normalized for cell numbers determined by nuclear DAPI counts in parallel samples) cellular fibronectin abundance in CDMs over time. **J)** Pairwise comparison of relative cellular fibronectin abundance in CDMs at each time point. Cellular fibronectin displayed fibrillar network in both MMC and nMMC CDMs, which became somewhat denser over time (**F**). Abundance of total fibronectin increased significantly between days 3 to 7, but remained unchanged after that in MMC CDMs. In contrast, total fibronectin levels were significantly increased up to 14 days in nMMC CDMs (**G and H**). In contrast to findings with cultures containing cells (Figure 5), there were no significant differences in relative (per cell) fibronectin levels over time in either CDM types (**I and J**). Pairwise comparison at each time point showed significantly higher relative fibronectin levels in MMC compared to nMMC CDMs (**J**), which was similar to cultures containing cells. Results show mean +/- SEM (**C, E, H, and J**) and statistical comparison over time (**B, D, G, and I**) from image analysis performed with 6-12 images obtained from each time point and treatment from two repeated experiments. Statistical testing was performed by one-way ANOVA (**B, D, G, and I**) and by independent samples t-test (**C, E, H, and J**), *p<0.05; **p<0.01; ***p<0.001. Magnification bars = 40 μm (**A and F**).

_
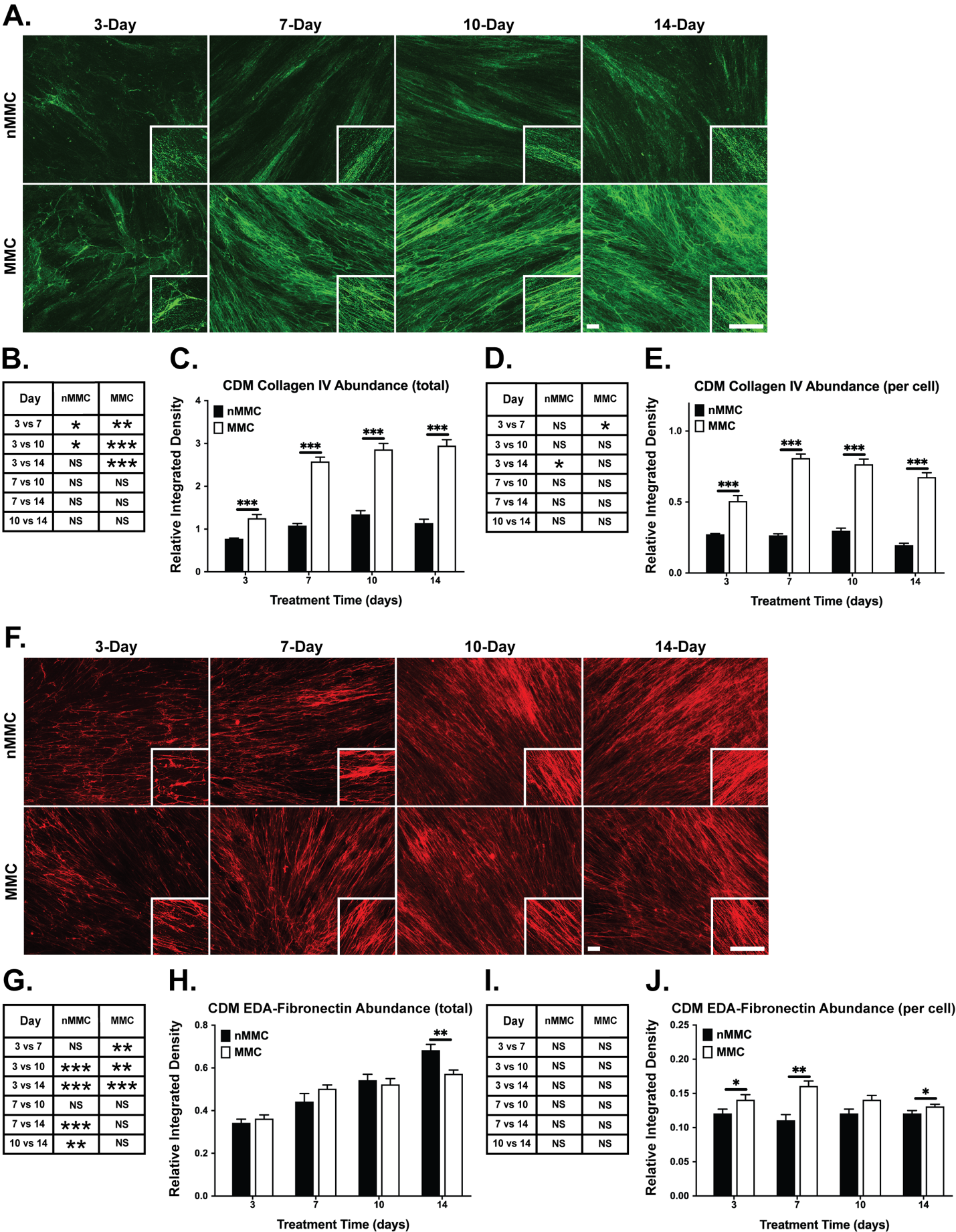
_

**Supplementary Figure S5. Characterization of laminin 1, tenascin C, SPARC, and LTBP1 organization and abundance in MMC and nMMC CDMs at day 14. A)** Representative standardized images of laminin 1, tenascin C, SPARC, and LTBP1 immunostaining from each time point and treatment from two repeated experiments. Magnification bar = 40 μm. Pairwise comparison of total (**B**) and relative (normalized for cell numbers determined by nuclear DAPI counts in parallel samples) (**C**) abundance of laminin 1, tenascin C, SPARC, and LTBP1 in CDMs at day 14. Laminin 1 and LTBP1 displayed better developed fibrillar network in CDMs from MMC compared to nMMC cultures, while organization of tenascin C and SPARC in both CDMs did not show marked differences (**A**). Similar to 14-day cultures containing cells (Figure 6), CDMs generated with MMC showed significantly higher levels of total and relative laminin 1 and tenascin C, relative levels of LTBP1, and lower levels of total and relative SPARC compared to nMMC CDMs (**B and C**). Results show mean +/- SEM from image analysis performed with 6-12 images obtained from each time point and treatment from two repeated experiments **(B and C)**. Statistical testing was performed by independent samples t-test, **p<0.01; ***p<0.001.


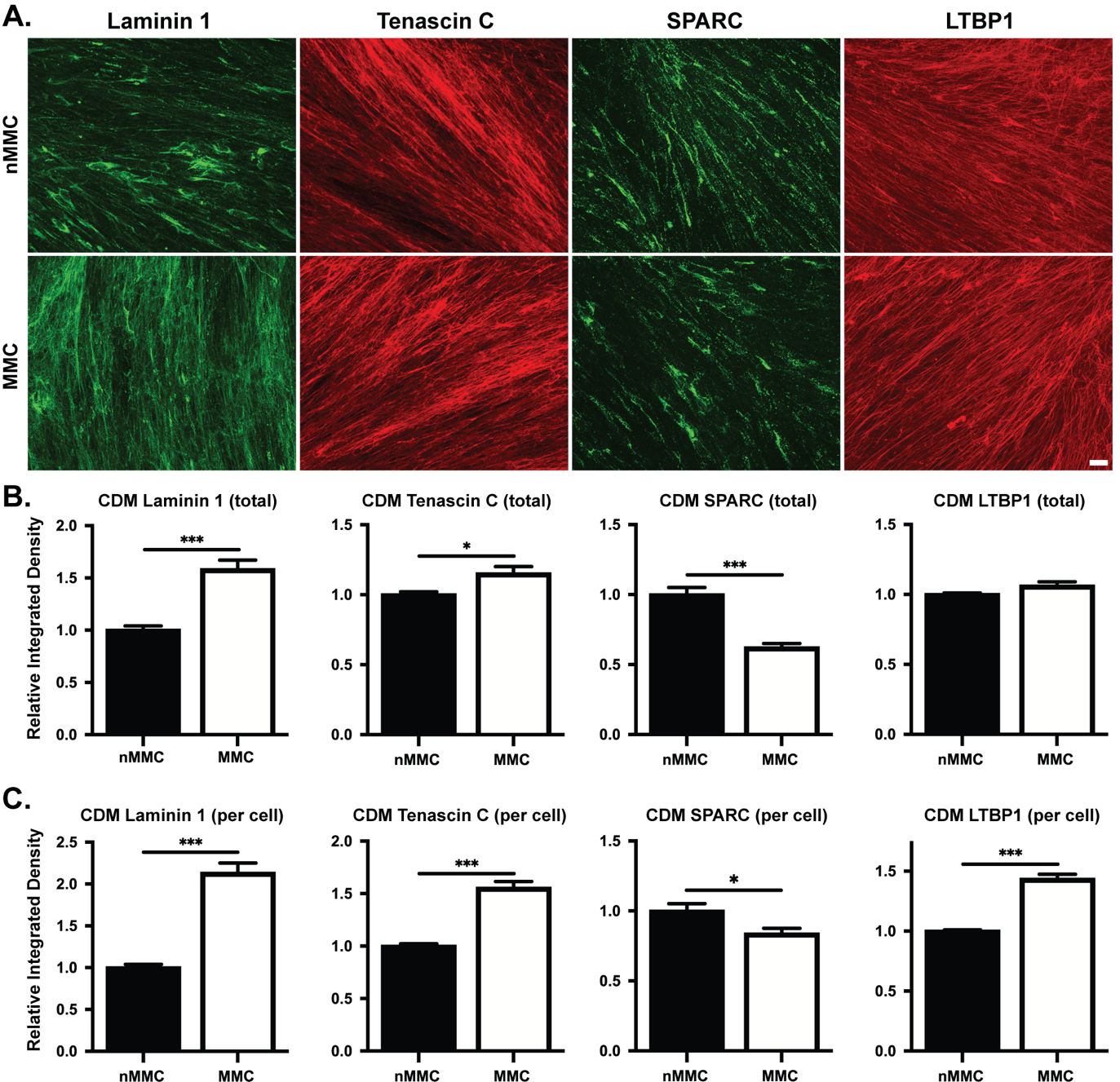


**Supplementary Figure S6. Assessment of ECM molecule loss in CDMs following decellularization of MMC and nMMC cultures. A)** Statistical comparison of change in total collagen I loss in CDMs over time. **B)** Pairwise comparison of total collagen I loss in CDMs at each time point. Both MMC and nMMC cultures lost collagen I (**A and B**). However, collagen I loss was reduced significantly in both MMC and nMMC cultures with increasing culture time (**A and B**). Pairwise comparison at each timepoint showed that cultures generated with MMC lost significantly less collagen I compared to nMMC cultures, ranging from 42% to 13% in MMC and 55% to 30% in nMMC CDMs at 3 to 14-day time points, respectively (**B**). **C)** Statistical comparison of change in total collagen IV loss in CDMs over time. **D)** Pairwise comparison of total collagen IV loss in CDMs at each time point. In contrast to collagen I, collagen IV showed increasing but statistically insignificant loss with increasing culture time in MMC cultures, ranging from less than 4% at day 3 to about 28% at later time points (**C and D**). Collagen IV loss in nMMC cultures increased significantly, peaking at day 7 (about 35% loss) (**C and D**). Pairwise comparison showed that both MMC and nMMC cultures displayed equal collagen IV loss, except at day 7 where nMMC cultures lost a significantly higher amount of collagen IV (**D**). **E)** Statistical comparison of change in total cellular fibronectin loss in CDMs over time. **F)** Pairwise comparison of total cellular fibronectin loss in CDMs at each time point. Decellularization caused also marked reduction of cellular fibronectin levels in both MMC and nMMC cultures with a small but significant decrease of loss over time from about 83% to 72% from day 3 to 14, respectively **(E and F)**. Pairwise comparison showed no significant difference in fibronectin loss between MMC and nMMC cultures at any timepoint (**F**). **G)** Pairwise comparison of tenascin C (loss of about 19%), SPARC (about 53%) and LTBP1 (about 75%), which were assessed in 14-day cultures, showed also protein loss after decellularization that was quantitatively similar in both MMC and nMMC cultures. In contrast, loss of laminin 1 was significantly lower in MMC (about 57%) compared to nMMC cultures (about 66%). Results show mean +/- SEM (**B, D, F, and G**) and statistical comparison over time (**A, C, and E**) from image analysis performed with 6-12 images obtained from each time point and treatment from two repeated experiments. Statistical testing was performed by one-way ANOVA (**A, C, and E**) and by independent samples t-test (**B, D, F, and G**), *p<0.05; **p<0.01; ***p<0.001.


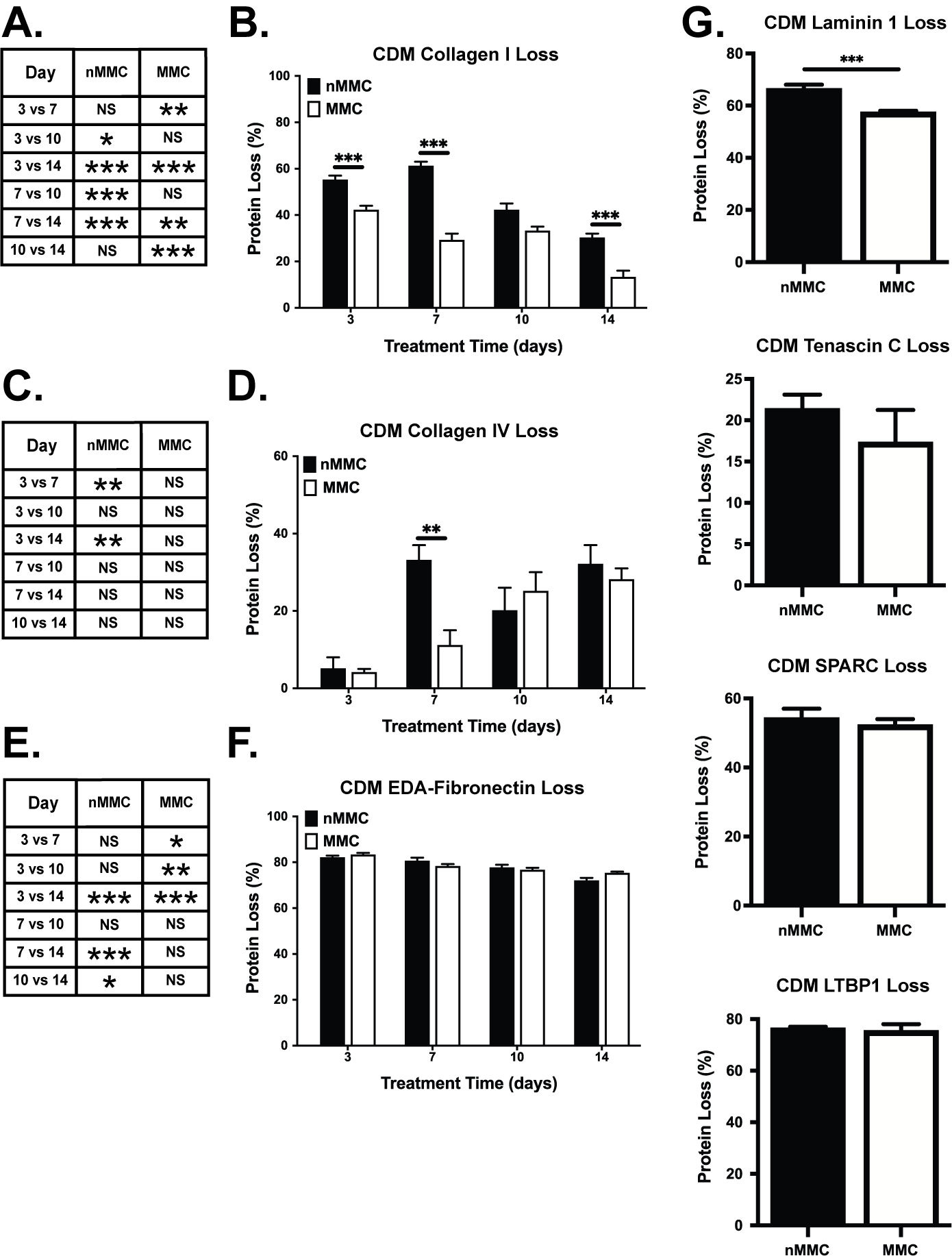


**Additional Information**

**Uncropped images of αSMA and GADPH Western blots shown in Figure 7B.**


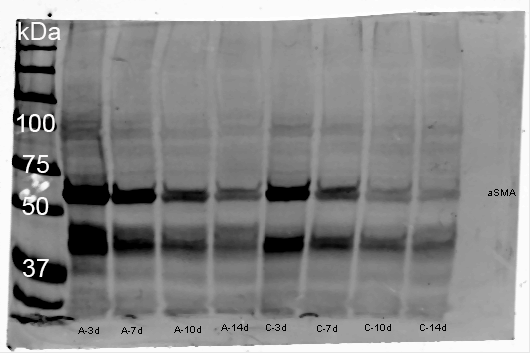

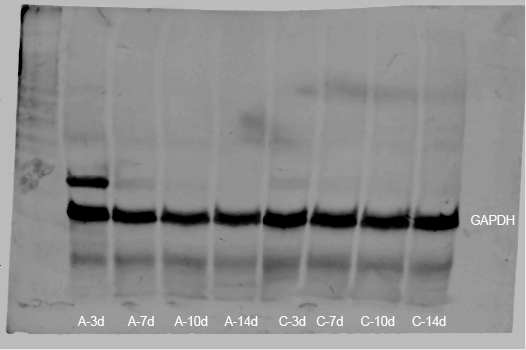

Supplement: Supplementary file 1 — Supplementary Information. [file 41598_2023_29252_MOESM1_ESM.docx]
